# Mechanism of Mechanical Activation-Assisted Selective Decalcification of Vanadium-Bearing Steel Slag

**DOI:** 10.3390/molecules31203596

**Published:** 2026-10-09

**Authors:** Minhao Zhang, Zekun Wang, Xuan Liu, Ang Zhao, Le Wang, Jinglong Liang

**Affiliations:** 1 College of Metallurgy and Energy, North China University of Science and Technology, Tangshan 063210, China; zhangminhao1415@163.com (M.Z.); wangzekun2333@163.com (Z.W.); 15903427492@163.com (X.L.); 15110759912@163.com (A.Z.); ljl@ncst.edu.cn (J.L.); 2 Yanzhao Iron and Steel Laboratory, North China University of Science and Technology, 21 Bohai Street, Tangshan 063210, China

**Keywords:** vanadium-bearing steel slag, mechanical activation, selective decalcification, kinetics, decalcification mechanism

## Abstract

Selective decalcification of vanadium-bearing steel slag via hydrochloric acid pretreatment was investigated in this work. The influences of hydrochloric acid concentration, temperature, reaction time, and liquid-to-solid ratio on Ca and V leaching from mechanically activated and non-activated slag are evaluated. The results show that mechanical activation significantly enhances decalcification selectivity. At an HCl concentration of 1.5 mol·L^−1^, 40 °C, 8 min, and a liquid-to-solid ratio of 7 mL·g^−1^, the Ca extraction reached 77.10%, while the V loss rate remains at only 6.08%, resulting in a separation index (*SI*) of 12.7, significantly higher than that of the non-activated slag (*SI* = 3.9). XRD and SEM-EDS analyses show the disappearance of Ca_2_SiO_4_, CaO, and Fe_3_O_4_ after decalcification, accompanied by the formation of a loose and porous surface structure. Ca_2_SiO_4_ preferentially dissolves due to its porous structure, whereas Ca_2_Fe_2_O_5_ remains because of its dense layered structure. Furthermore, the lower bond energy of Ca-O than V-O facilitates selective decalcification. ICP analysis shows that the CaO content decreases from 40.83% to 19.87%, while the V_2_O_5_ content increases from 2.19% to 2.49%, resulting in vanadium enrichment. Kinetic analysis suggests a shrinking-core model for the decalcification process, with the reaction being predominantly governed by surface chemical control. The apparent reaction order and activation energy are 0.73 and 20.28 kJ·mol^−1^ respectively. These findings provide theoretical support for selective calcium removal and vanadium enrichment from vanadium-bearing steel slag.

## 1. Introduction

Driven by the rapid expansion of the aerospace, railway, and automotive sectors, the global demand for vanadium products has increased steadily in recent years. As an important strategic metal, vanadium plays a critical role in a variety of industrial applications. Its earliest and most extensive use is in steel production, where the addition of vanadium enhances mechanical properties by refining grain structure and modifying the microstructure [1]. Benefiting from its outstanding alloying characteristics, vanadium has also become an indispensable component in aerospace materials [2]. Beyond metallurgical applications, vanadium is extensively utilized in catalysts [3,4], energy storage systems [5], pharmaceuticals [6], and other advanced technologies. Commercial vanadium resources are primarily obtained from vanadium–titanium magnetite [7] and vanadium-bearing stone coal [8]. The processing of V-Ti magnetite inevitably produces vanadium-bearing steel slag. With the sustained growth of the steelmaking sector, the accumulation of this secondary resource continues to rise. Although the vanadium content in vanadium-bearing steel slag (1–5 wt.%) falls below that of converter slag, it still substantially exceeds stone coal in V content, making it a promising feedstock for vanadium recovery [9]. The utilization of vanadium-bearing steel slag is complicated by its heterogeneous composition and intricate mineralogical characteristics. Calcium- and iron-containing phases constitute the major components of the slag matrix [10], while elements such as Mg, P, V, and Si are distributed among various mineral phases. The abundance of calcium and iron not only increases processing complexity but also hinders the efficient extraction of vanadium. Consequently, developing effective approaches for reclaiming vanadium from V-bearing steelmaking slag has become an important topic of ongoing research.

Current technologies for reclaiming vanadium from V-bearing steelmaking slag can generally be categorized into pyrometallurgical and hydrometallurgical routes. Among them, roasting–leaching processes remain the dominant pyrometallurgical approach. Conventional roasting–leaching using sodium salts is still prevalent in industry, where sodium-containing additives such as Na_2_CO_3_ and NaOH are added to convert V-bearing phases into soluble sodium vanadate species at elevated temperatures, thereby facilitating subsequent vanadium extraction [11]. Despite its high extraction efficiency, this process generates large volumes of saline wastewater, posing significant environmental and disposal challenges [12]. To overcome these drawbacks, calcification roasting has attracted increasing attention as a more environmentally benign alternative. In this process, CaO or calcium-bearing additives are used as roasting agents to oxidize low-valence vanadium species in vanadium spinels and convert them into acid-soluble phases such as calcium vanadates. Compared with sodium roasting, calcification roasting avoids the emission of corrosive and polluting gases, including Cl_2_, HCl, and SO_2_, associated with sodium salts during high-temperature treatment. Moreover, its compatibility with calcium-rich vanadium-bearing steel slag makes it a relatively cleaner option for vanadium recovery.

Nevertheless, several limitations restrict the industrial application of Ca roasting. The conversion rate of V-bearing phases is often unsatisfactory, leading to relatively high processing costs and limited economic competitiveness [13]. In addition, when treating phosphorus-rich vanadium-bearing steel slag, stable solid solutions such as Ca_3_(PO_4_)_2_-Ca_2_SiO_4_ and complex encapsulated phases are readily formed during roasting. As a result, part of the vanadium becomes immobilized within phosphorus-containing calcium phases, which suppresses phase transformation and the subsequent leaching of vanadium, thereby reducing recovery efficiency [14]. Consequently, calcification roasting is not well suited to high-phosphorus vanadium-bearing steel slag and exhibits inherent application limitations.

In contrast, hydrometallurgical processes recover vanadium through direct leaching, with acid leaching currently being the most extensively investigated approach.

Direct acid leaching recovers vanadium by employing acidic media, such as sulfuric acid or hydrochloric acid, to directly disrupt the spinel structure of vanadium-bearing steel slag, thereby releasing vanadium from the solid matrix into the solution phase. Compared with conventional roasting–leaching processes, direct acid leaching avoids high-temperature roasting and offers several advantages, including a shorter process flow, lower energy consumption, the absence of roasting-related gas emissions, and simpler equipment requirements [15]. As a result, it is widely regarded as a promising environmentally friendly route for vanadium recovery.

Although direct acid leaching features a relatively simple process and a high vanadium leaching rate, its acid consumption remains substantial [16]. This is mainly associated with the preferential consumption of acid by CaO and other acid-consuming elements, including Fe, Cr, and Mg, contained in vanadium-bearing minerals. To overcome this limitation, several pretreatment approaches have been proposed prior to leaching. These approaches generally rely on physical or chemical methods, including the crushing, separation, and selective removal of specific elements, to alter the original phase structure of vanadium-bearing steel slag and thereby enhance the leaching rate of target elements in subsequent extraction processes.

Iron in vanadium-bearing steel slag mainly exists as metallic iron and iron-containing spinels, which exhibit distinct magnetic properties. Therefore, magnetic separation following crushing and grinding can be employed to recover iron-bearing phases while simultaneously enriching vanadium in the non-magnetic fraction, creating favorable conditions for subsequent vanadium extraction. Xiang et al. [17] investigated the phase composition and microstructure of converter slag and reported that iron was predominantly distributed in spinel and olivine phases. By combining grinding and screening followed by magnetic separation, approximately 41.5% of metallic iron was recovered, with an iron grade of 85%. Meanwhile, the non-magnetic product contained 8.56 wt.% V, corresponding to a recovery of 95.3%, while the Ti reached 8.63% with a recovery of 85.3%. The magnetic product is suitable for recycling into steelmaking, while the non-magnetic fraction serves as a suitable feedstock for further vanadium recovery.

To reduce the interference of calcium and iron with vanadium extraction, Gao et al. [18] proposed a selective pretreatment method for vanadium-bearing steel slag and investigated its decomposition behavior in a hydrochloric acid system. The effects of temperature, liquid-to-solid (L/S) ratio, acid concentration, and other operating variables in calcium dissolution were systematically examined. Under the optimal conditions of an HCl concentration of 2 mol·L^−1^,40 °C, a reaction time of 10 min, a particle size range of 71–124 μm, and an L/S ratio of 8 mL·g^−1^, the CaO content decreased from 41.09% to 14.28%. This pretreatment effectively mitigated the influence of calcium on subsequent vanadium leaching and demonstrated considerable industrial potential.

Pretreatment technologies can significantly reduce acid consumption and minimize the interference of impurity elements during leaching, thereby improving the leaching rate of target elements. However, vanadium loss inevitably occurs during pretreatment, and the underlying mechanism governing this phenomenon remains insufficiently understood. Therefore, further optimization of pretreatment strategies is still required.

In addition to pretreatment technologies, mechanical activation through ball milling has also attracted attention as an effective approach for enhancing the leaching rate of target elements. As a high-energy activation technique, ball milling can substantially modify the microstructure of steel slag and improve the reactivity of its constituent phases. By introducing additional lattice defects and increasing the stored surface energy, mechanical activation promotes the utilization of steel slag in cement production, hydrometallurgical extraction, energy-related applications, and resource recovery processes.

Mechanical activation is an effective approach for improving the physicochemical properties of steel slag. Ali Allahverdi et al. [19] mechanically activated high-phosphorus steel slag using a high-performance ball mill and evaluated the mechanical response of the activated slag under varying fineness levels. Their results showed that values as high as 93 MPa after 28 days could be achieved at a fineness of 450 m^2^·kg^−1^, indicating that ball milling effectively enhances the mechanical properties of steel slag.

Similarly, S. K. Singh et al. [20] modified steel slag through a mechano-chemical activation process and observed significant changes in the morphology of hydration products after activation. Their study suggested that ball milling for 20 min effectively improves the reactivity of steel slag. At the microstructural level, Elena Lysenko et al. [21] activated Fe_2_O_3_-Li_2_CO_3_-ZnO mixtures using a planetary high-energy ball mill and employed XRD and SEM-EDS analyses to characterize the resulting structural transformation. The results demonstrated that ball milling reduces particle size and enhances material reactivity. These findings provide valuable insights into the mechanism of mechanical activation, indicating that ball milling can significantly alter microstructures and temporarily store energy within distorted crystal lattices, thereby improving material performance. Collectively, the above findings highlight the considerable promise of mechanical activation in enabling effective utilization and recovery of valuable resources from steel slag.

This study investigates a selective decalcification process employing mechanical activation and hydrochloric acid leaching for vanadium-bearing steel slag. The selective removal of calcium from mechanically activated and non-activated slag is systematically evaluated under various operating conditions. Process parameters are optimized, and a separation index (*SI*) is introduced to quantitatively assess decalcification performance and identify the optimal operating conditions. The phase evolution, microstructural characteristics, and surface chemical states before and after decalcification are examined by XRD, SEM-EDS, and XPS. Kinetic modeling is employed to determine the rate-limiting step during decalcification process. From a chemical bond perspective, the mechanism underlying selective calcium removal with vanadium retention is elucidated, providing theoretical support for both the efficient pretreatment of vanadium-bearing steel slag and the recovery of vanadium resources.

## 2. Results and Discussion

### 2.1. Thermodynamic Analysis

**Figure 1:**
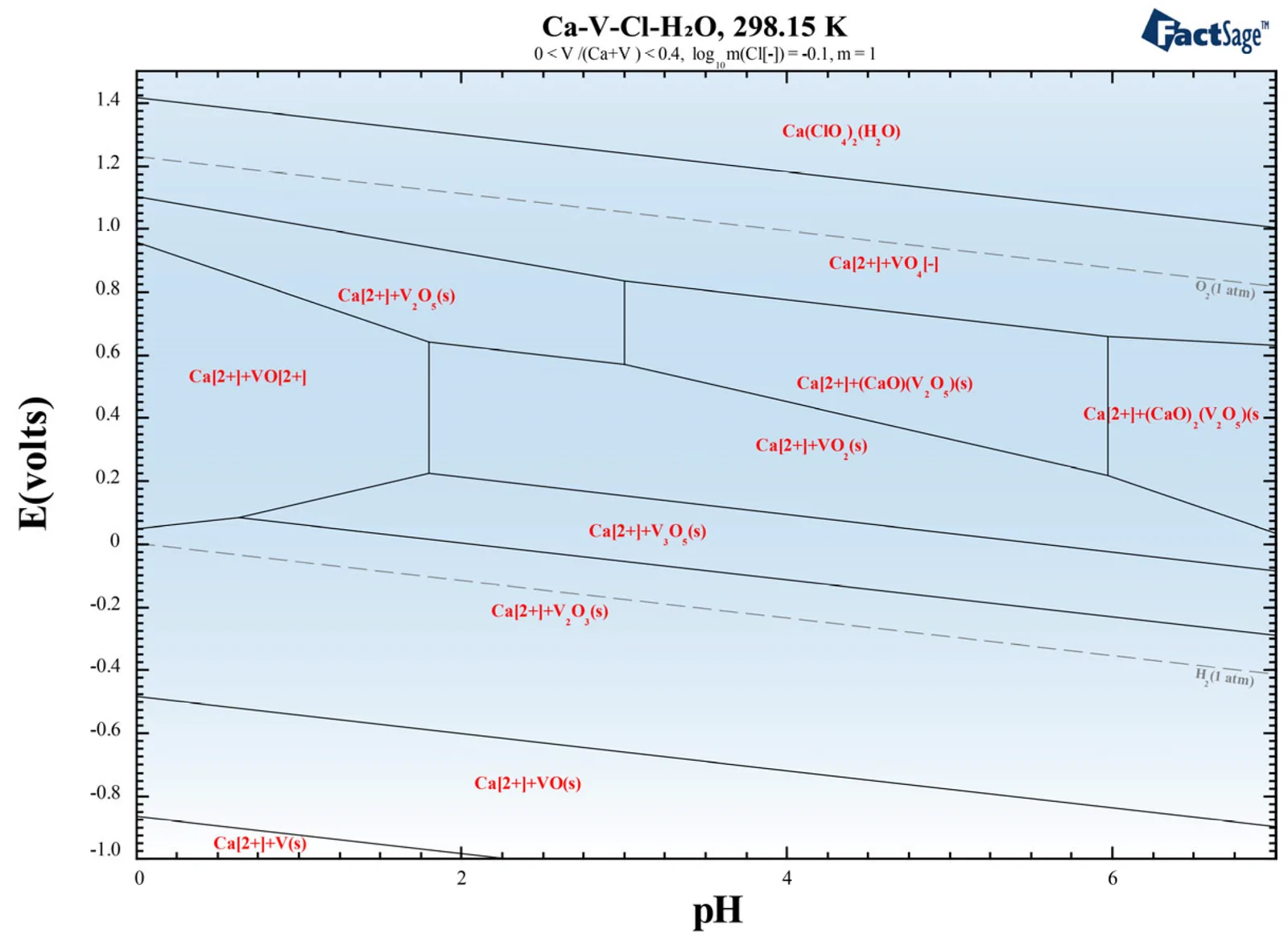
E-pH diagram of the Ca-V-Cl-H_2_O system.

**Figure 2:**
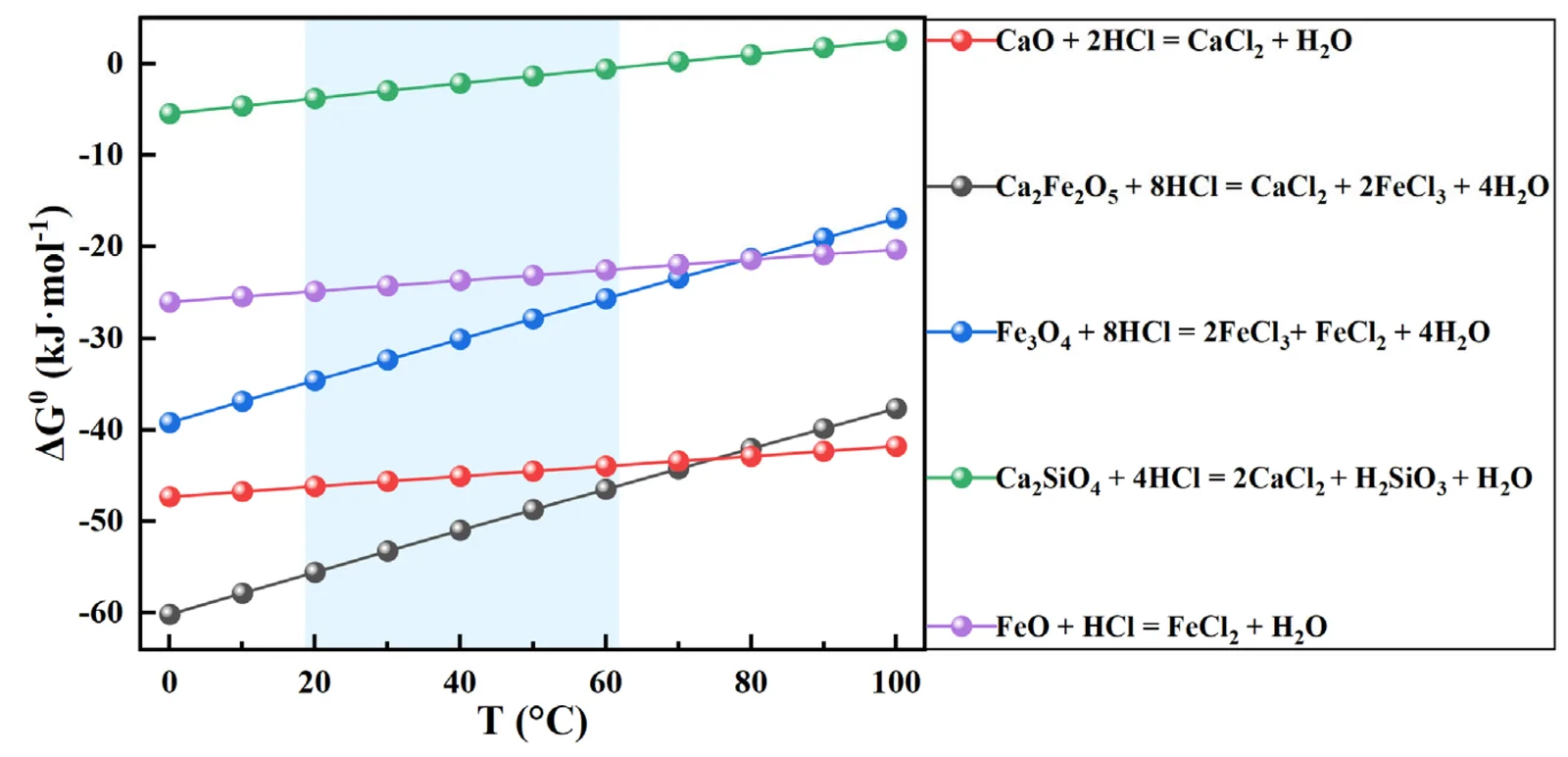
Relationship between Δ*G*^0^ and temperature.

To systematically evaluate the thermodynamic behavior of the decalcification process and clarify the speciation of Ca and V during hydrochloric acid leaching, an E-pH diagram of the Ca-V-Cl-H_2_O system was constructed using FactSage 6.4 at 298.15 K and atmospheric pressure (Figure 1). The calculation was performed with a total metal molality of 1 mol·kg^−1^, a vanadium mole fraction of 0 < V/(Ca + V) < 0.4, and log_10_m(Cl^−^) = −0.1. Relevant aqueous and solid Ca-, V-, Cl-, and H-containing species available in FactSage 6.4 were considered. Thermodynamic equilibrium was assumed, while kinetic effects were neglected. Within the investigated acidic range, calcium predominantly exists as soluble Ca^2+^ and readily enters the solution, whereas vanadium remains stable in the solid phase. This distinct thermodynamic behavior enables the selective separation of calcium from vanadium, thereby achieving the goal of decalcification with vanadium retention. To further assess the thermodynamic feasibility of decalcification, the Gibbs free energy changes of the relevant reactions in the hydrochloric acid leaching system were evaluated using HSC Chemistry 6.0. The reactions considered are listed in Figure 2 and the corresponding relationships between Δ*G*^0^ and temperature are presented in Figure 2. The thermodynamic data were obtained from the database integrated in HSC Chemistry 6.0. Over the investigated temperature range, all reactions involving calcium-bearing phases exhibit negative Gibbs free energy values, indicating that these reactions are thermodynamically feasible. Consequently, calcium is dissolved as Ca^2+^, confirming the feasibility of selective decalcification. Notably, impurity elements such as Fe are also co-dissolved to varying extents.

### 2.2. Effects of Process Conditions on Selective Decalcification

The effects of hydrochloric acid concentration, reaction temperature, reaction time, and L/S ratio on the decalcification process were systematically investigated. Ca is the primary leached element, and the leaching behavior of V directly determines the process selectivity. All tests were conducted in triplicate, and the reported values are the arithmetic means of three independent experiments. Error bars for V and Ca leaching rates denote one standard deviation (SD, *n* = 3), reflecting the consistency and reproducibility of the experimental data. Results for impurity elements are presented as mean values only to illustrate their leaching trends.

#### 2.2.1. Effect of HCl Concentration on Selective Decalcification

**Figure 3:**
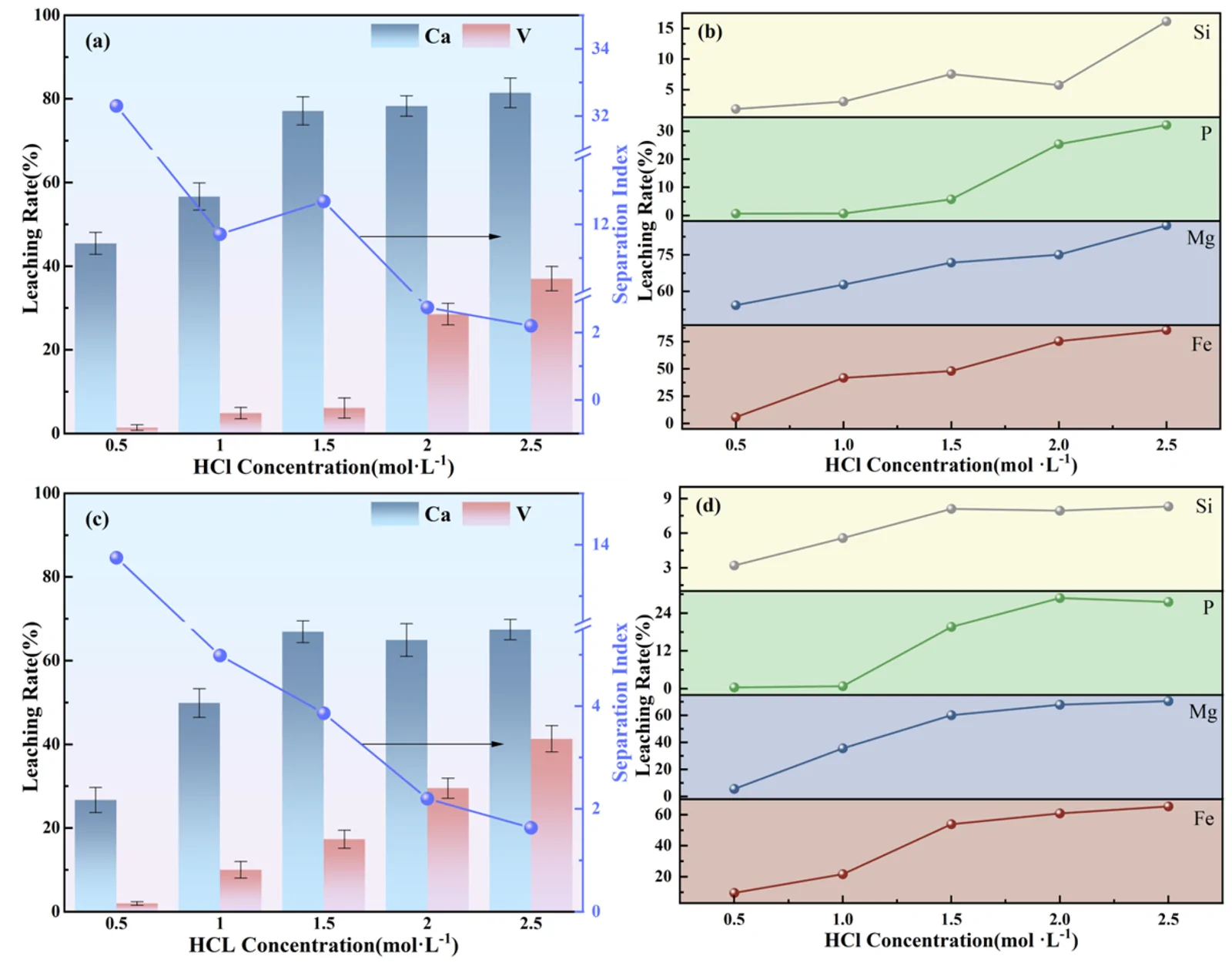
Leaching rates of various elements at different HCl concentrations: (**a**) leaching rates of Ca and V from the mechanically activated slag; (**b**) leaching rates of other elements from the mechanically activated slag; (**c**) leaching rates of Ca and V from the non-activated slag; (**d**) leaching rates of other elements from the non-activated slag (temperature: 40 °C, time: 8 min, liquid-to-solid ratio: 7 mL·g^−1^).

The effect of HCl concentration on selective decalcification was investigated under fixed conditions of an L/S ratio of 7 mL·g^−1^, 8 min, and 40 °C. The HCl concentration was varied from 0.5 to 2.5 mol·L^−1^ with an interval of 0.5 mol·L^−1^. The corresponding leaching rates of the major elements are presented in Figure 3.

Figure 3a,c show the leaching behaviors of Ca and V as a function of HCl concentration. For the mechanically activated slag (Figure 3a), the Ca leaching rate rose from 45.42% at 0.5 mol·L^−1^ to 81.42% at 2.5 mol·L^−1^, whereas the V leaching rate remained relatively low (1.41–37.00%). Accordingly, the *SI* decreased from 32.3 to 2.2 with increasing acid concentration. It is noteworthy that both Ca and V exhibited relatively low leaching rates at 0.5 mol·L^−1^ HCl. Although a high *SI* value was obtained under this condition, the low Ca leaching rate limited the practical significance of the selectivity evaluation. When the HCl concentration reached 2.0 mol·L^−1^ or higher, excessive proton activity substantially reduced selectivity and resulted in significant vanadium co-leaching. In contrast, the non-activated slag (Figure 3c) exhibited lower Ca leaching rates but higher V leaching rates than the mechanically activated slag under identical conditions. Moreover, the *SI* values of the non-activated slag (1.6–13.7) were consistently lower than those of the activated slag, demonstrating that mechanical activation markedly enhances the selectivity of the decalcification process. Figure 3b,d present Fe, Mg, P, and Si leaching behaviors from the activated and non-activated slags, respectively. The leaching rates of all four elements increased with increasing HCl concentration, indicating enhanced dissolution under stronger acidic conditions.

**Figure 4:**
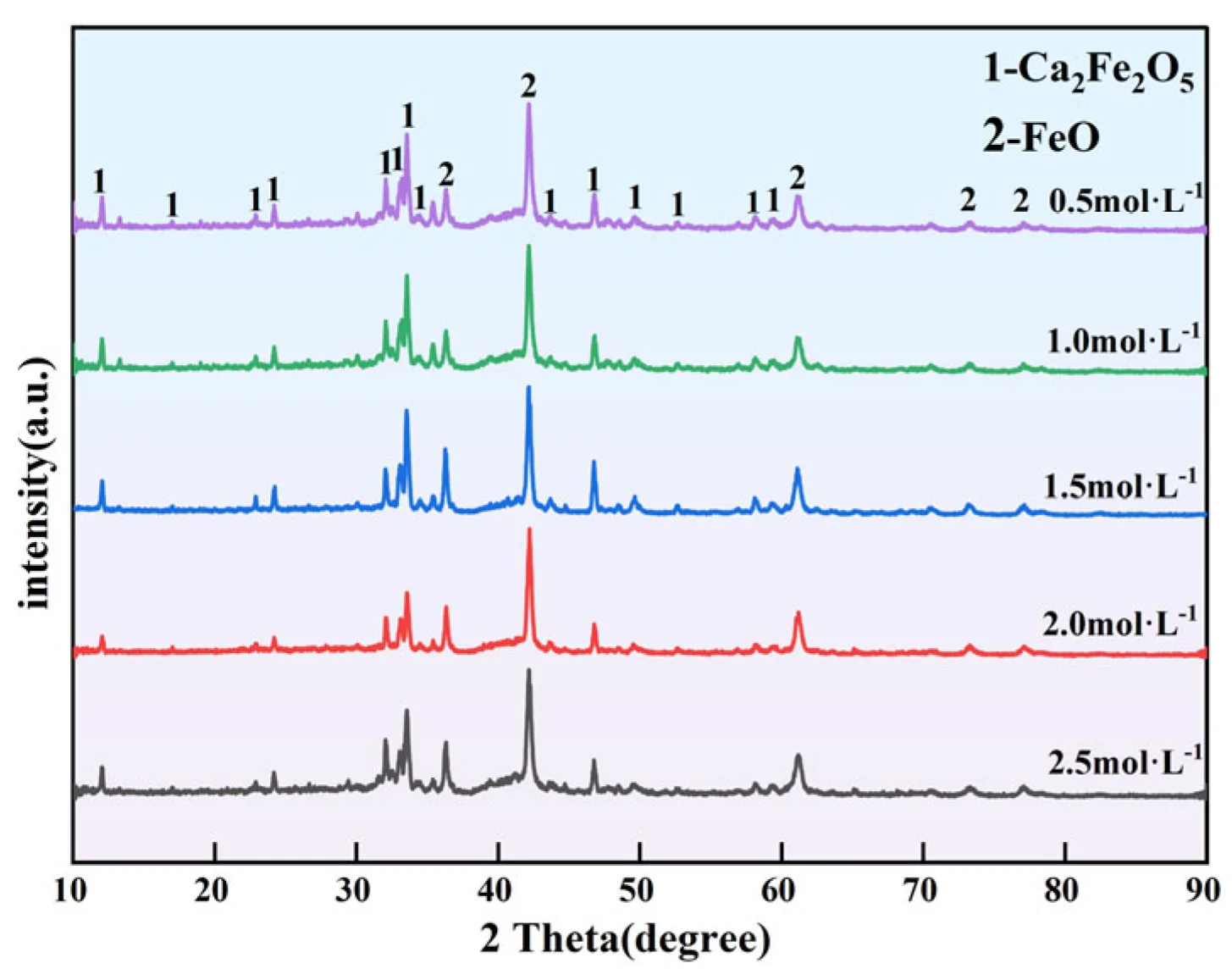
XRD patterns of mechanically activated slag at different HCl concentrations.

To further investigate the phase evolution during decalcification, the products obtained from mechanically activated slag at different HCl concentrations were analyzed using XRD, as shown in Figure 4. Only C_2_F and FeO were detected in all decalcified products, whereas the diffraction peaks of the calcium-bearing phases C_2_S and CaO, together with Fe_3_O_4_ present in the raw slag, were no longer detected after treatment, possibly because their contents fell below the XRD detection limit or they transformed into amorphous phases. This phase evolution confirms the feasibility of the pre-decalcification process. Calcium in calcium-bearing phases such as C_2_S is preferentially dissolved by hydrochloric acid, whereas the C_2_F phase remains stable in the residue. As a result, an initial separation of calcium and vanadium is achieved, creating favorable conditions for subsequent vanadium recovery.

Considering both the *SI* values and the actual leaching rates of Ca and V, a moderate HCl concentration provides an effective balance between efficient calcium removal and the suppression of excessive vanadium dissolution. At low acid concentrations, decalcification is limited by insufficient acid supply, whereas excessive acidity significantly reduces selectivity. Therefore, the optimal HCl concentration was identified to be 1.5 mol·L^−1^.

#### 2.2.2. Effect of Reaction Temperature on Selective Decalcification

**Figure 5:**
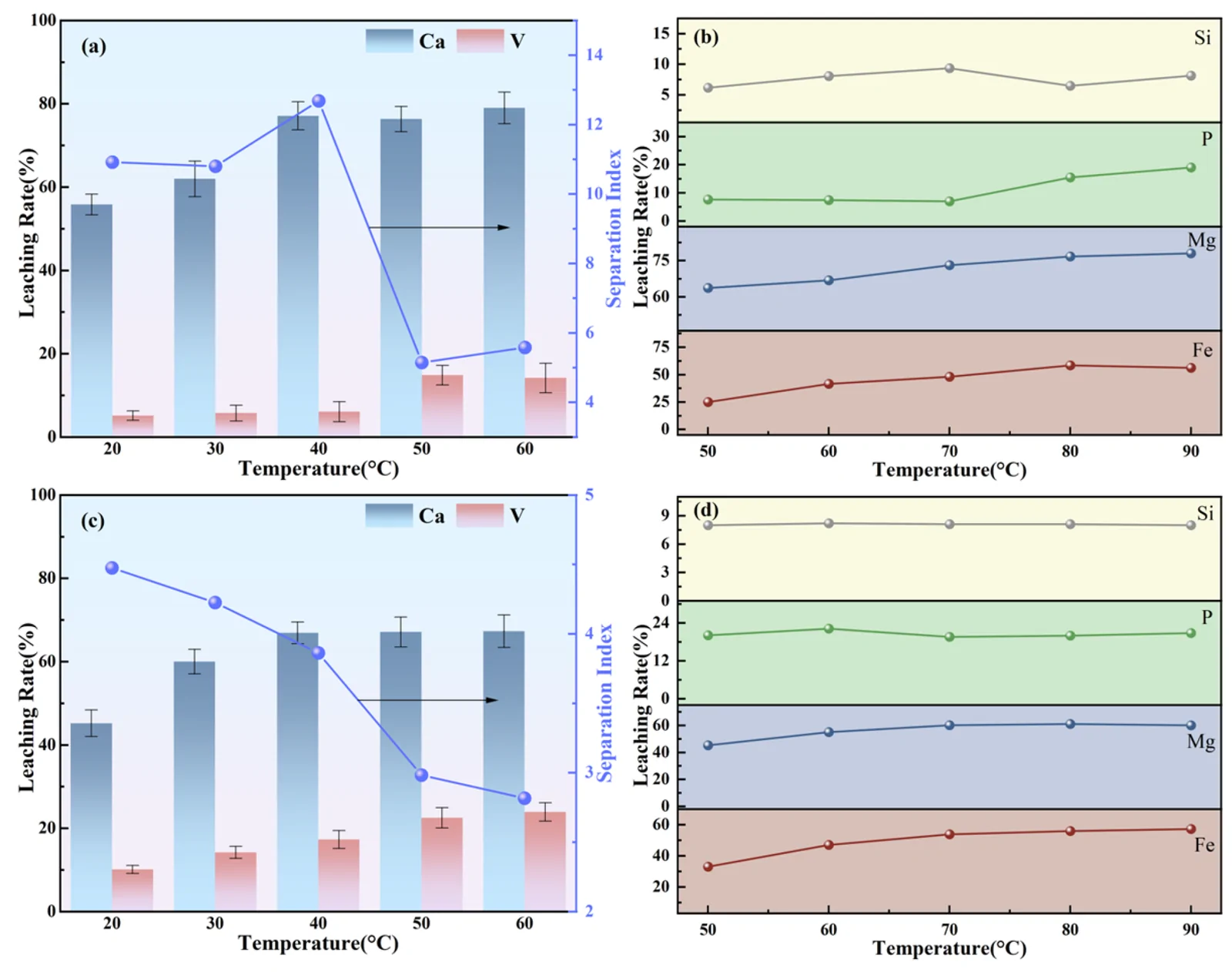
Leaching rates of various elements at different reaction temperatures: (**a**) leaching rates of Ca and V from the mechanically activated slag; (**b**) leaching rates of other elements from the mechanically activated slag; (**c**) leaching rates of Ca and V from the non-activated slag; (**d**) leaching rates of other elements from the non-activated slag (HCl concentration: 1.5 mol·L^−1^, time: 8 min, liquid-to-solid ratio: 7 mL·g^−1^).

The effect of reaction temperature on selective decalcification was evaluated at a fixed HCl concentration of 1.5 mol·L^−1^, a reaction time of 8 min, and an L/S ratio of 7 mL·g^−1^. The reaction temperature ranged between 20 and 60 °C (10 °C intervals). The corresponding leaching rates of the major elements are presented in Figure 5.

Figure 5a,c show how temperature affects Ca and V leaching behaviors. For the mechanically activated slag (Figure 5a), the Ca leaching rate rose from 55.82% at 20 °C to 77.10% at 40 °C. By contrast, the V leaching rate increased only gradually with increasing temperature and remained at a relatively low level (≤6.08%). The enhanced calcium dissolution at elevated temperatures is attributed to accelerated reaction kinetics and improved mass transfer. When the temperature exceeded 40 °C, the Ca leaching rate gradually approached a plateau, whereas the V leaching rate continued to increase. Consequently, the *SI* increased initially and decreased thereafter, peaking at 12.7 at 40 °C. The temperature-dependent leaching behaviors of Ca and V in the non-activated slag are shown in Figure 5c. Similar trends were observed compared with those of the mechanically activated slag; however, the Ca leaching rates were consistently lower, whereas the V leaching rates were higher. Accordingly, the *SI* decreased from 4.5 to 2.8 with increasing temperature, remaining substantially lower than that of the activated slag. Figure 5b,d present Fe, Mg, P, and Si leaching behaviors from the activated and non-activated slags, respectively. Fe, Mg, and P leaching rates increased with increasing temperature, whereas that of Si remained relatively stable throughout the investigated temperature range.

**Figure 6:**
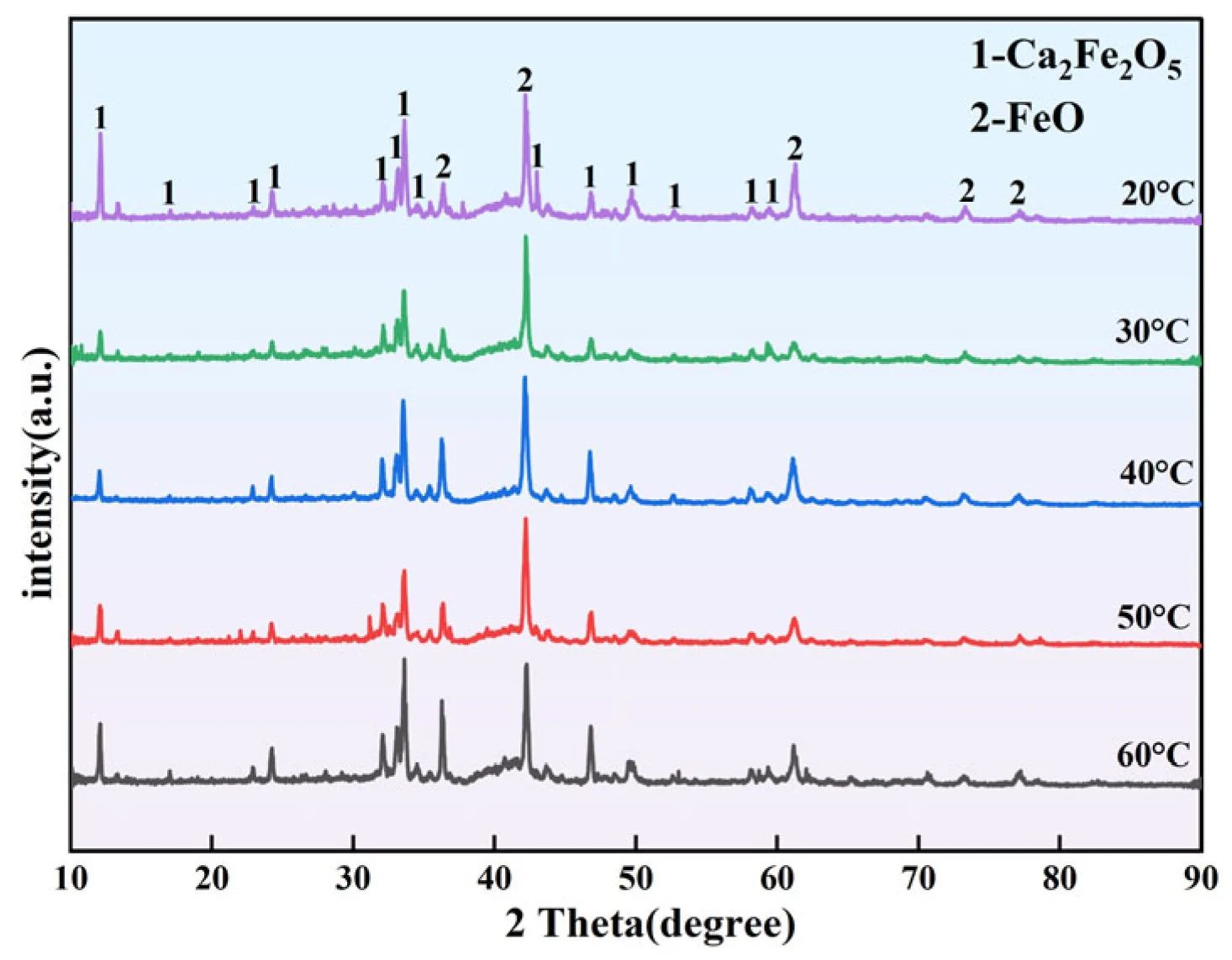
XRD patterns of mechanically activated slag at different temperatures.

To further investigate the phase evolution during decalcification, the products obtained from mechanically activated slag at different temperatures were characterized by XRD, as shown in Figure 6. Only C_2_F and FeO were detected in all decalcified products, whereas no residual C_2_S, CaO, or Fe_3_O_4_ phases were observed, possibly because their contents decreased below the XRD detection limit or they transformed into amorphous phases. This result indicates that calcium-bearing phases such as C_2_S were substantially removed within the investigated temperature range. Although increasing temperature accelerated the decalcification process, no noticeable change in phase composition was observed, indicating that temperature primarily affected the reaction rate rather than the selective separation pathway.

Overall, increasing temperature within an appropriate range promoted calcium dissolution. However, excessively high temperatures also facilitated non-selective vanadium leaching. Therefore, considering both leaching efficiency and selectivity, the reaction temperature of 40 °C was selected as optimal.

#### 2.2.3. Effect of Reaction Time on Selective Decalcification

**Figure 7:**
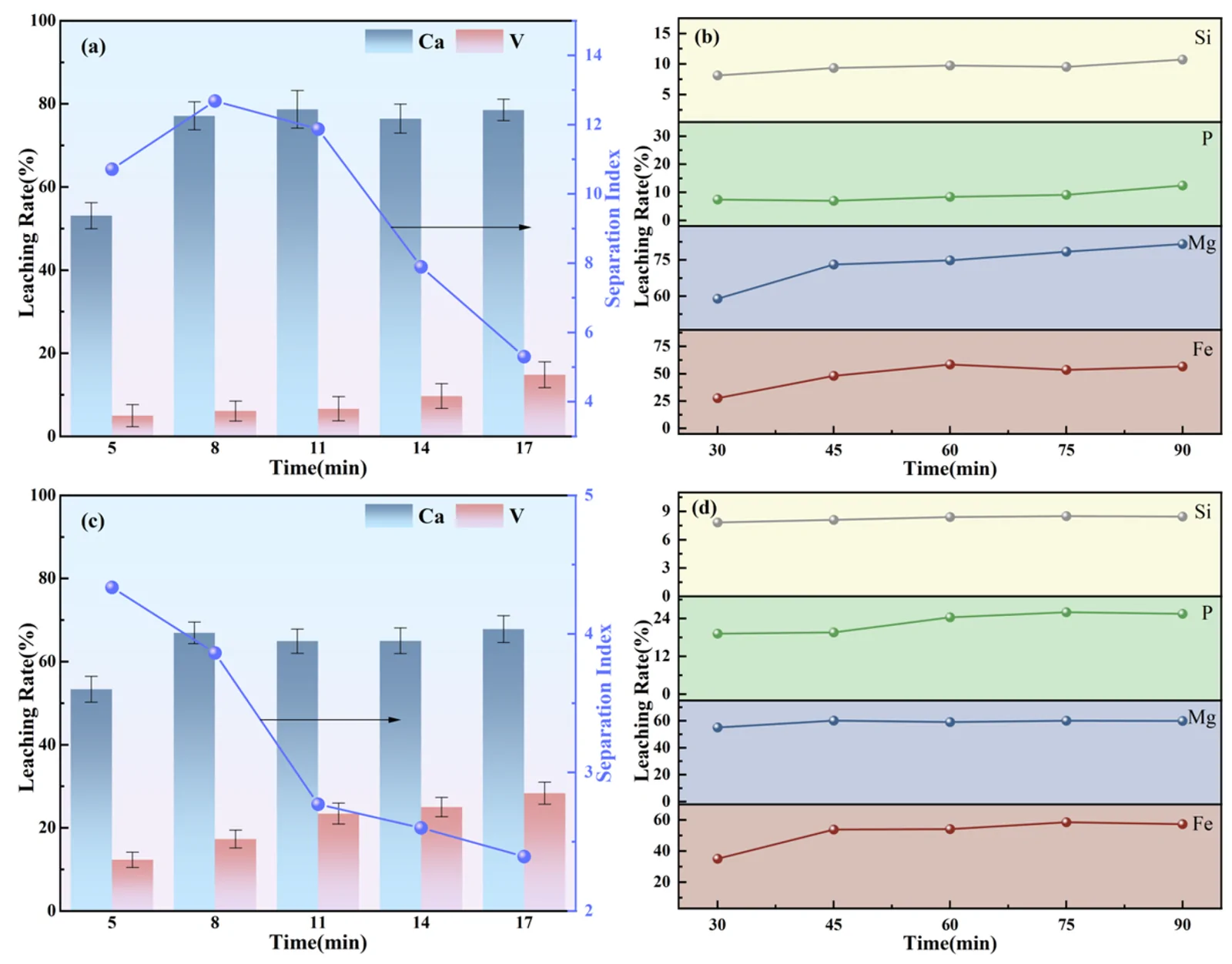
Leaching rates of various elements at different reaction times: (**a**) leaching rates of Ca and V from the mechanically activated slag; (**b**) leaching rates of other elements from the mechanically activated slag; (**c**) leaching rates of Ca and V from the non-activated slag; (**d**) leaching rates of other elements from the non-activated slag (HCl concentration: 1.5 mol·L^−1^, temperature: 40 °C, liquid-to-solid ratio: 7 mL·g^−1^).

The effect of reaction time on selective decalcification was investigated at 40 °C, a fixed HCl concentration of 1.5 mol·L^−1^, and an L/S ratio of 7 mL·g^−1^. The reaction time was varied from 5 to 17 min with an interval of 3 min. The major element leaching rates are presented in Figure 7.

Figure 7a,c show how reaction time affects the leaching behaviors of Ca and V. For the mechanically activated slag (Figure 7a), Ca extraction rose rapidly during the initial stage of leaching and reached 77.10% within 8 min, followed by a gradual increase toward a plateau. This behavior indicates that the readily soluble calcium-bearing phases were preferentially dissolved in the initial reaction stage, after which the process became increasingly constrained by diffusion and structural limitations. In contrast, the V leaching rate remained at a relatively low level (≤6.08%) during this period but increased to 14.81% after 14 min. Accordingly, the *SI* peaked at 12.7 at 8 min and subsequently dropped to 5.3 as Ca dissolution gradually leveled off while V continued to leach into solution. These results suggest that decalcification mainly occurred during the initial stage of the reaction, whereas excessive prolongation of the reaction time promoted non-selective vanadium dissolution and reduced process selectivity. As shown in Figure 7c, the Ca leaching rates of the non-activated slag remained consistently lower than those of the mechanically activated slag throughout the investigated time range. Correspondingly, the *SI* values were also lower than those obtained for the activated slag. Figure 7b,d present the leaching behaviors of Fe, Mg, P, and Si from the activated and non-activated slags, respectively. Fe and Mg leaching rates increased with increasing reaction time, whereas those of Si and P gradually approached stable values.

**Figure 8:**
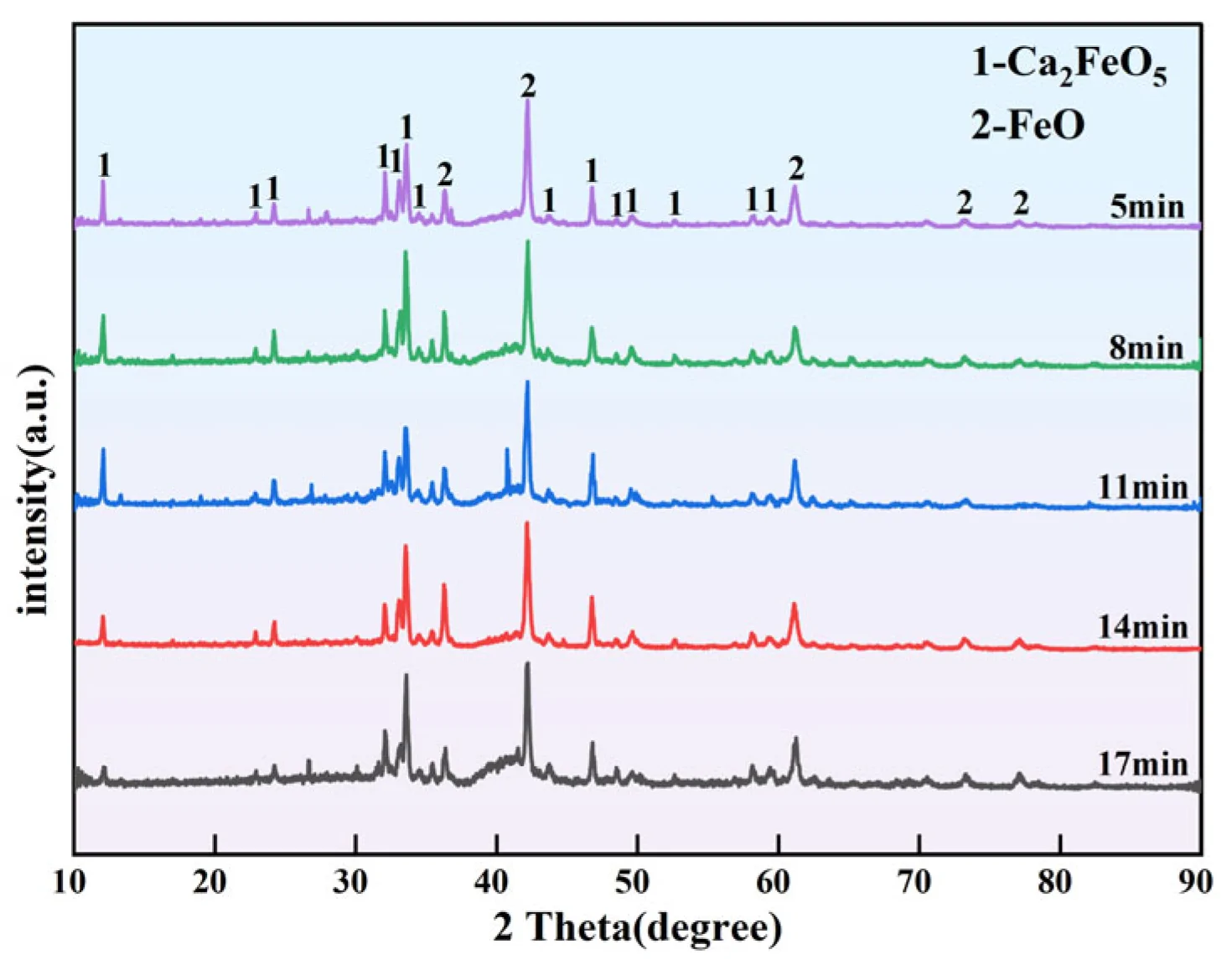
XRD patterns of mechanically activated slag at different reaction times.

To further investigate the phase evolution during decalcification, the products obtained from mechanically activated slag at different reaction times were characterized by XRD, as shown in Figure 8. Only C_2_F and FeO were detected in the decalcified products, even after a relatively short reaction time of 5 min. These results indicate that calcium-bearing phases such as C_2_S were effectively removed during the early stage of leaching. Although prolonged reaction time improved the extent of calcium dissolution, the phase composition of the decalcified residue remained unchanged.

Considering both the *SI* values and the leaching rates of Ca and V, a reaction time of 8 min provided the optimal balance between decalcification efficiency and selectivity. Shorter reaction times resulted in insufficient calcium dissolution due to mass-transfer limitations, whereas prolonged leaching promoted continuous vanadium dissolution and reduced selectivity. Therefore, 8 min was selected as the optimal reaction time.

#### 2.2.4. Effect of Liquid-to-Solid Ratio on Selective Decalcification

**Figure 9:**
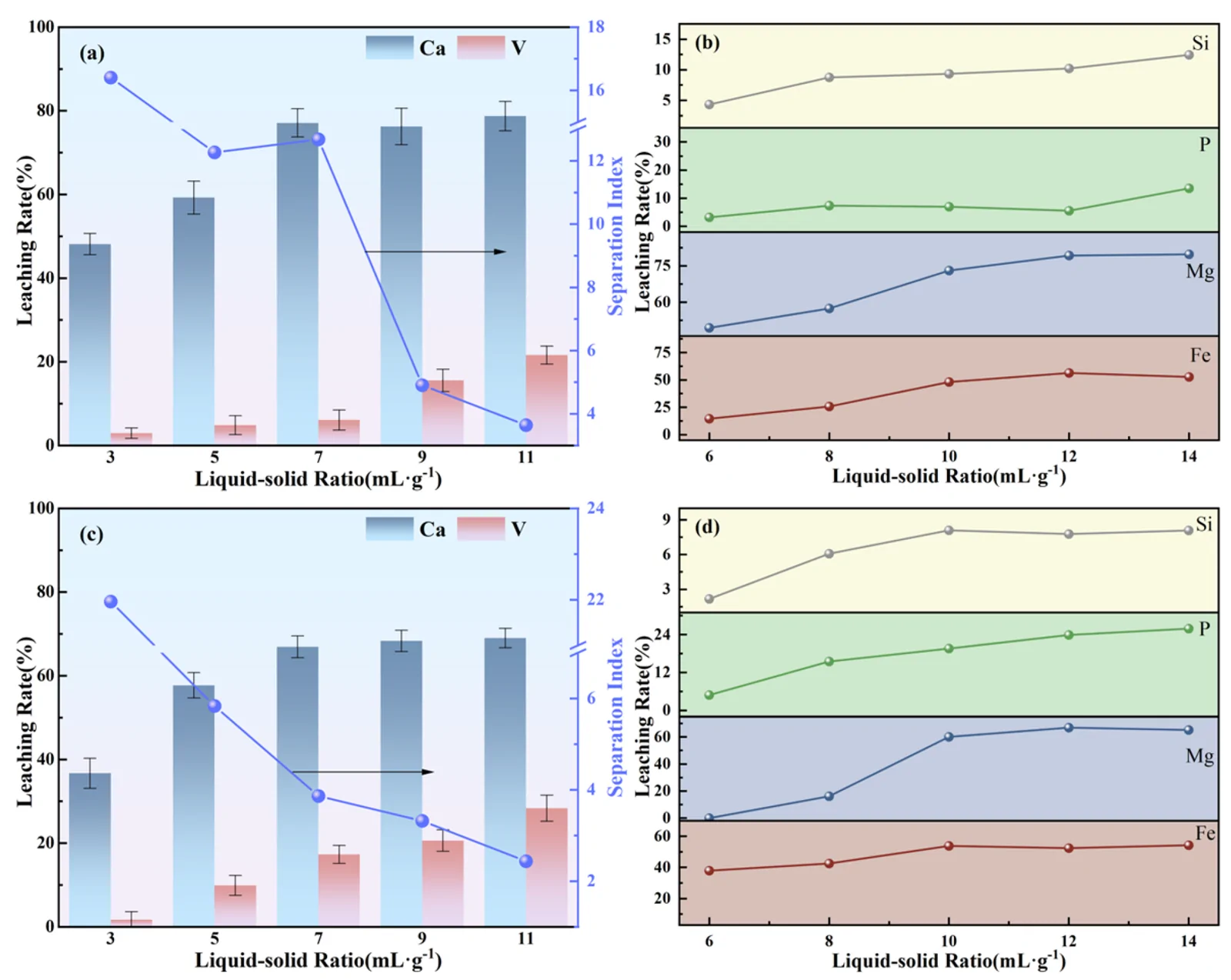
Leaching behaviors of various elements at different liquid-to-solid ratios: (**a**) leaching rates of Ca and V from the mechanically activated slag; (**b**) leaching rates of other elements from the mechanically activated slag; (**c**) leaching rates of Ca and V from the non-activated slag; (**d**) leaching rates of other elements from the non-activated slag.

The experimental conditions were fixed at a reaction time of 8 min, a temperature of 40 °C, and an HCl concentration of 1.5 mol·L^−1^, while the L/S ratio was varied from 3 mL·g^−1^ to 11 mL·g^−1^ at intervals of 2 to assess selective decalcification. The corresponding leaching rates of the investigated elements are presented in Figure 9.

Figure 9a,c illustrate the effect of the L/S ratio on Ca and V leaching behaviors. For the mechanically activated slag (Figure 9a), increasing the L/S ratio from 3 mL·g^−1^ to 7 mL·g^−1^ significantly enhanced the Ca leaching rate from 48.14% to 77.10%, whereas the V leaching rate remained relatively low (2.93–6.08%). Correspondingly, the *SI* initially decreased from 16.4 to 12.3 and then increased slightly to 12.7. At an L/S ratio of 3 mL·g^−1^, both Ca and V leaching were subject to pronounced mass-transfer limitations; because the restricted dissolution affected Ca and V to a comparable extent, the *SI* exhibited an artificially elevated value that did not accurately reflect the intrinsic selectivity of the system. As the L/S ratio increased further, the Ca leaching rate gradually approached a plateau while the V leaching rate continued to rise, causing the *SI* to stagnate or even decrease slightly. This trend indicates that the readily soluble calcium-bearing phases had been largely depleted and the system was approaching equilibrium. Meanwhile, the increased liquid volume improved mass-transfer conditions and facilitated the continued dissolution of the residual solid phases, thereby enhancing vanadium release. Consequently, the leaching gap between Ca and V was progressively narrowed, preventing further improvement in selectivity.

For the non-activated slag (Figure 9c), the leaching trends of Ca and V were qualitatively similar to those of the activated slag, but the Ca leaching rates remained consistently lower and the corresponding *SI* values were markedly inferior. Without mechanical activation, the calcium-bearing phases retained a more compact and stable structure that resisted effective disruption by hydrochloric acid, thereby limiting preferential calcium dissolution. Simultaneously, this less effective structural disruption also rendered vanadium more susceptible to co-release during leaching, further diminishing selectivity. This comparison underscores the essential role of mechanical activation in enabling selective decalcification.

Figure 9b,d present the leaching behaviors of Fe, Mg, P, and Si in the activated and non-activated slags, respectively. Both Fe and Mg exhibited increasing leaching rates as the L/S ratio increasing, indicating that a larger liquid volume was favorable for the dissolution of these elements.

**Figure 10:**
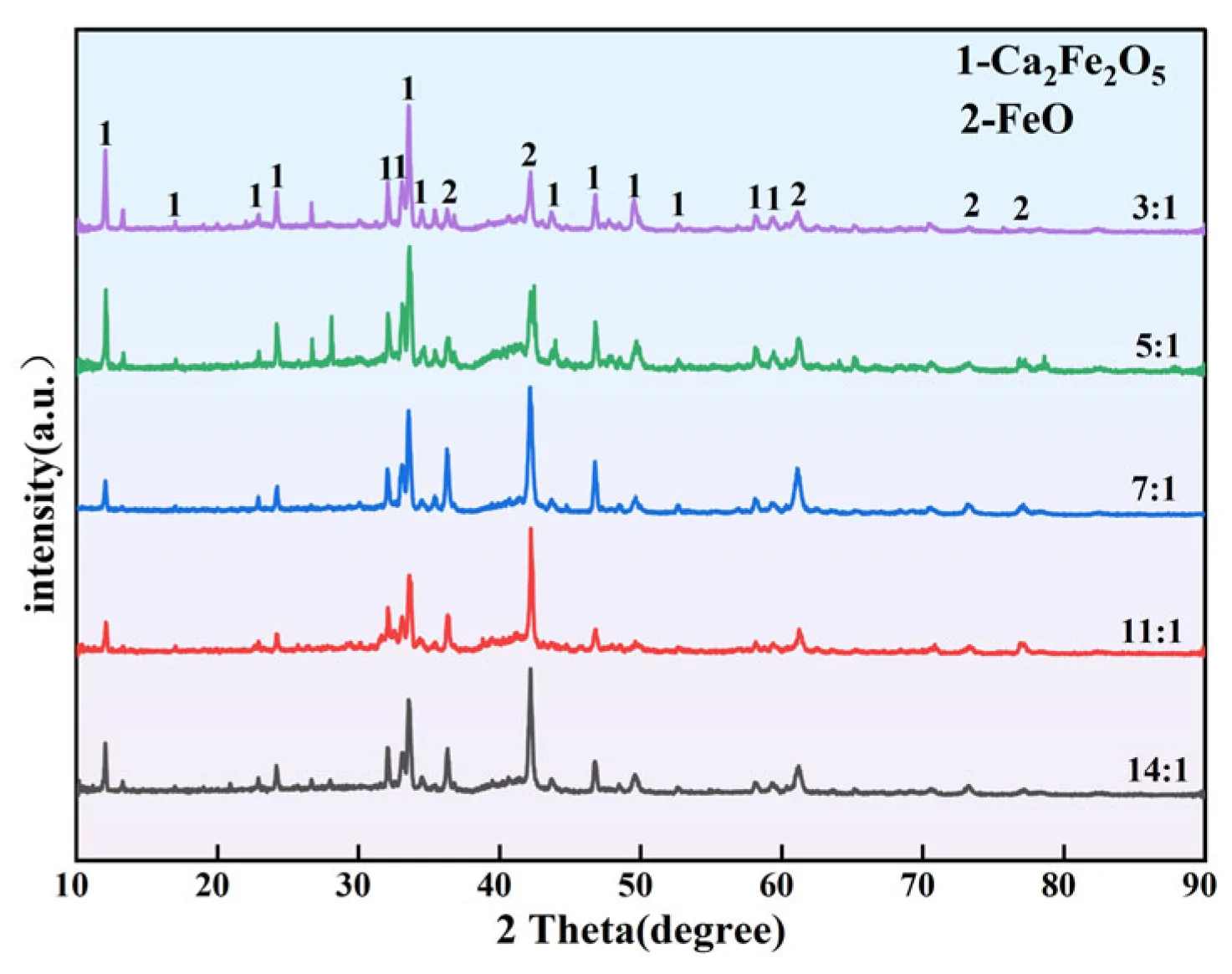
XRD patterns of mechanically activated slag at different liquid-to-solid ratios.

The solid residues obtained at different L/S ratios were characterized by XRD to investigate phase evolution, as shown in Figure 10. The diffraction patterns reveal that all decalcified products contained only C_2_F and FeO, with no detectable residual signals from the original calcium-bearing phases (C_2_S, CaO) or Fe_3_O_4_. Even at the lowest L/S ratio of 3 mL·g^−1^, where solid–liquid mass transfer was relatively restricted, C_2_S and CaO were completely dissolved. Further increases in the L/S ratio did not alter the phase composition of the products, indicating that this parameter primarily governed mass-transfer efficiency rather than the final phase assemblage.

Taking into account the *SI* values and the actual Ca and V leaching rates, an L/S ratio of 7 mL·g^−1^ achieved the most favorable balance between decalcification efficiency and selectivity. Compared with the insufficient leaching caused by mass-transfer limitations at lower L/S ratios and the decline in selectivity arising from continued vanadium dissolution at higher ratios, this condition maintained a high Ca leaching rate while effectively suppressing excessive vanadium release. Therefore, 7:1 mL·g^−1^ was selected as the optimal L/S ratio.

Overall, under the optimal conditions of an HCl concentration of 1.5 mol·L^−1^, an L/S ratio of 7 mL·g^−1^, a reaction time of 8 min, and a temperature of 40 °C, the mechanically activated slag achieved a Ca leaching rate of 77.10%, a V loss rate of only 6.08%, and a maximum *SI* of 12.7, which was significantly superior to the decalcification performance of the non-activated slag in parallel experiments (Ca leaching rate of 66.90% and *SI* = 3.9). The limited further increase in Ca leaching under more intensive conditions indicates that the decalcification process gradually approached a plateau after the preferential dissolution of readily leachable Ca-bearing phases. Since the objective of this study was selective decalcification rather than complete Ca removal, excessively severe leaching conditions were not preferred because they could increase vanadium co-leaching and reduce the separation selectivity. This enhancement can be attributed to ball-milling-induced structural disorder and lattice deformation [22], which disrupt the compact structure of the slag and expose additional calcium-bearing sites. Consequently, the reactive interface between hydrochloric acid and the slag increased, enhancing the leaching kinetics of calcium-bearing phases such as C_2_S and promoting their preferential dissolution while largely preserving the structural integrity of vanadium-bearing sites [23].

The optimized process parameters identified through the single-factor experiments provide an experimental foundation for the subsequent mechanistic investigation of the decalcification process.

### 2.3. Characterization of Decalcified Products

To determine the elemental makeup of the pre-decalcified slag obtained under optimal leaching conditions (L/S ratio of 7 mL·g^−1^, temperature of 40 °C, HCl concentration of 1.5 mol·L^−1^, and reaction time of 8 min), the residue was digested and quantitatively analyzed by ICP. The compositional profiles of the raw vanadium-bearing steel slag and the resulting pre-decalcified residue are compared in Table 1. CaO underwent a marked reduction from 40.83 wt.% to 19.87 wt.%, whereas the V_2_O_5_ content increased slightly from 2.19 wt.% to 2.49 wt.%. This indicates that the primary effect of the pre-decalcification treatment was the selective removal of Ca rather than substantial vanadium enrichment. Other impurity elements also exhibited varying degrees of relative enrichment.

**Figure 11:**
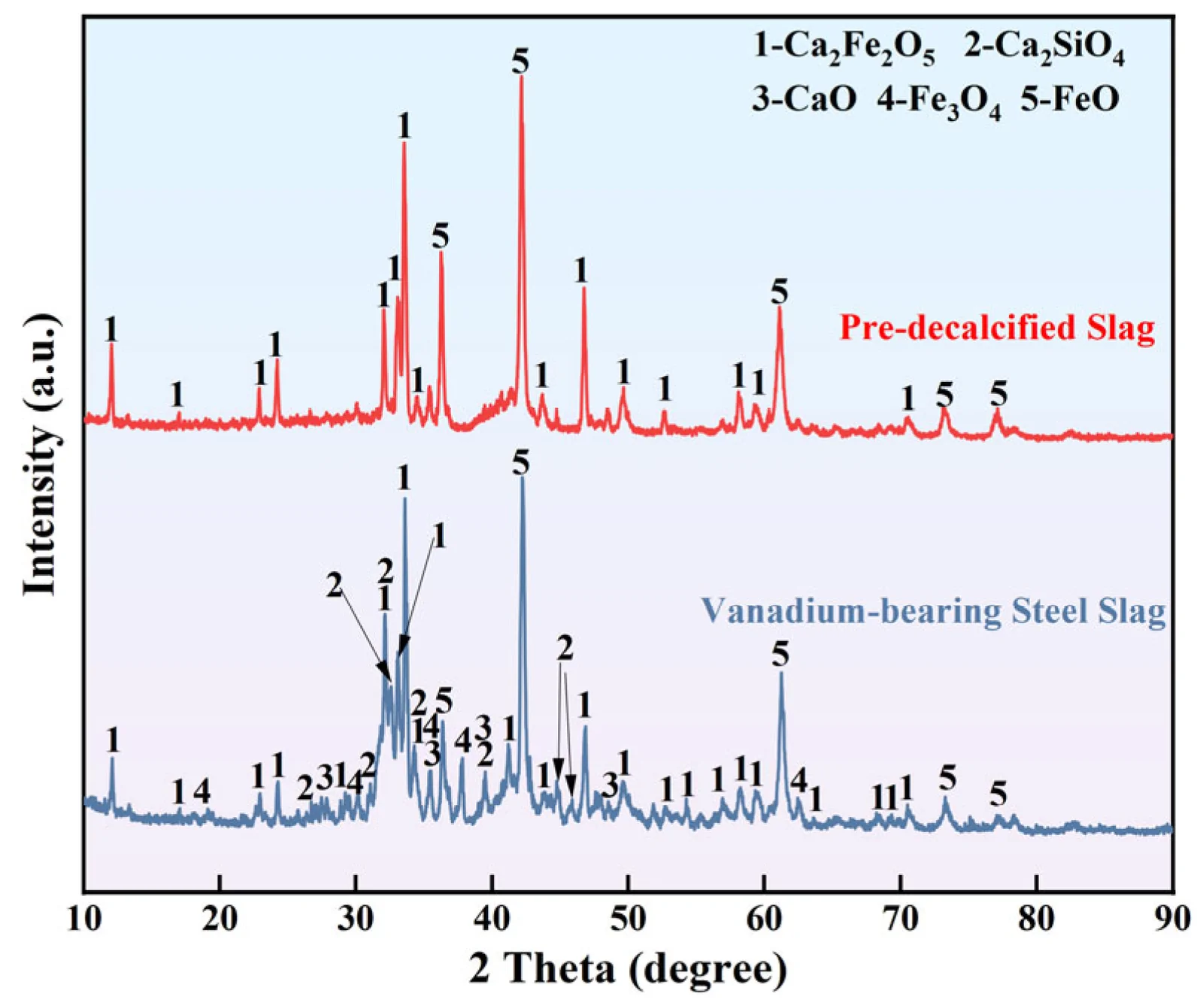
XRD patterns of vanadium-bearing steel slag and pre-decalcified slag.

Figure 11 compares the XRD profiles of the raw slag and the residue after decalcification. After decalcification, the characteristic peaks of CaO, Fe_3_O_4_, and Ca_2_SiO_4_ were markedly weakened, possibly because their contents decreased below the XRD detection limit or they transformed into amorphous phases, whereas Ca_2_Fe_2_O_5_ and FeO remained as the dominant phases. These results indicate that readily soluble calcium-bearing phases preferentially dissolve during hydrochloric acid leaching, while part of the iron oxide phases may undergo dissolution or structural transformation under acidic conditions.

The preferential dissolution of Ca_2_SiO_4_ is closely related to its crystal structure. C_2_S possesses a chain-like silicate framework composed of CaO_x_ polyhedra and isolated SiO_4_ tetrahedra linked through shared oxygen atoms. Owing to its relatively open structure, Ca^2+^ ions are distributed within interstitial lattice sites and are more susceptible to proton attack. Under acidic conditions, H^+^ preferentially attacks Ca-O-Si linkages, resulting in bond cleavage, lattice breakdown, and the subsequent release of Ca^2+^ into solution.

In contrast, Fe^3+^ in C_2_F forms stable Fe-O polyhedral units with relatively high coordination numbers, which are interconnected through shared vertices and edges to create a compact three-dimensional framework. This structure contains strong Fe-O bonds, while Ca^2+^ is incorporated within a comparatively stable coordination environment, rendering the phase less susceptible to acid attack. In addition, an Fe-rich residual skeleton tends to form during leaching, further hindering solution penetration into the particle interior and reducing the overall dissolution rate. Consequently, C_2_F exhibits considerably greater resistance to dissolution and becomes enriched in the solid residue.

Previous studies have reported that silicate minerals undergo decalcification more readily in acidic media and exhibit substantially higher dissolution rates than ferrite phases [24]. Therefore, the differences in structural stability and dissolution behavior between C_2_S and C_2_F are responsible for the preferential dissolution of silicate phases and the relative retention of ferrite phases during the decalcification process.

**Figure 12:**
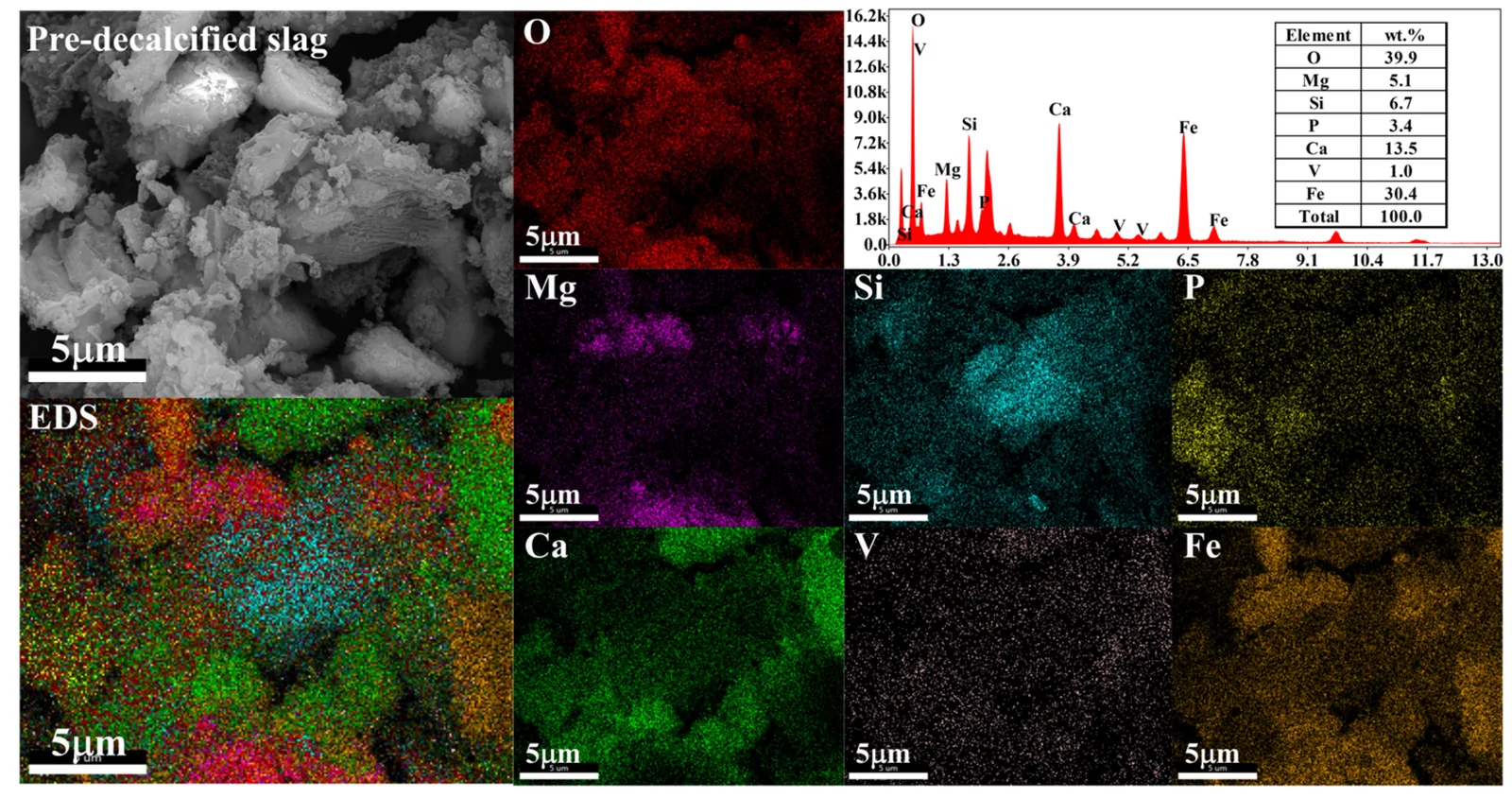
SEM image and EDS elemental mapping of pre-decalcified slag.

To further investigate the microstructural evolution and elemental distribution of the pre-decalcified slag, SEM-EDS characterization was performed. The SEM image and corresponding elemental mapping results are presented in Figure 12. Compared with the raw slag, the decalcified residue exhibits a noticeably looser structure with distinct dissolution features in localized regions, indicating the preferential dissolution of calcium-bearing phases during hydrochloric acid leaching.

The elemental mapping results show a substantial reduction in the abundance of Ca and a less uniform distribution after decalcification. Residual Ca is mainly concentrated in peripheral regions. In contrast, Fe becomes relatively enriched and exhibits a more continuous distribution in certain areas, suggesting that the original calcium-bearing phases were preferentially removed while Fe-rich phases were largely retained. Silicon also shows a certain degree of enrichment, which is likely associated with the persistence of silicate framework residues following calcium removal.

Vanadium remains broadly dispersed throughout the residue but exhibits partial spatial overlap with Fe- and Si-rich regions. This distribution pattern suggests that vanadium undergoes little migration during leaching and remains predominantly associated with the residual solid phases.

**Figure 13:**
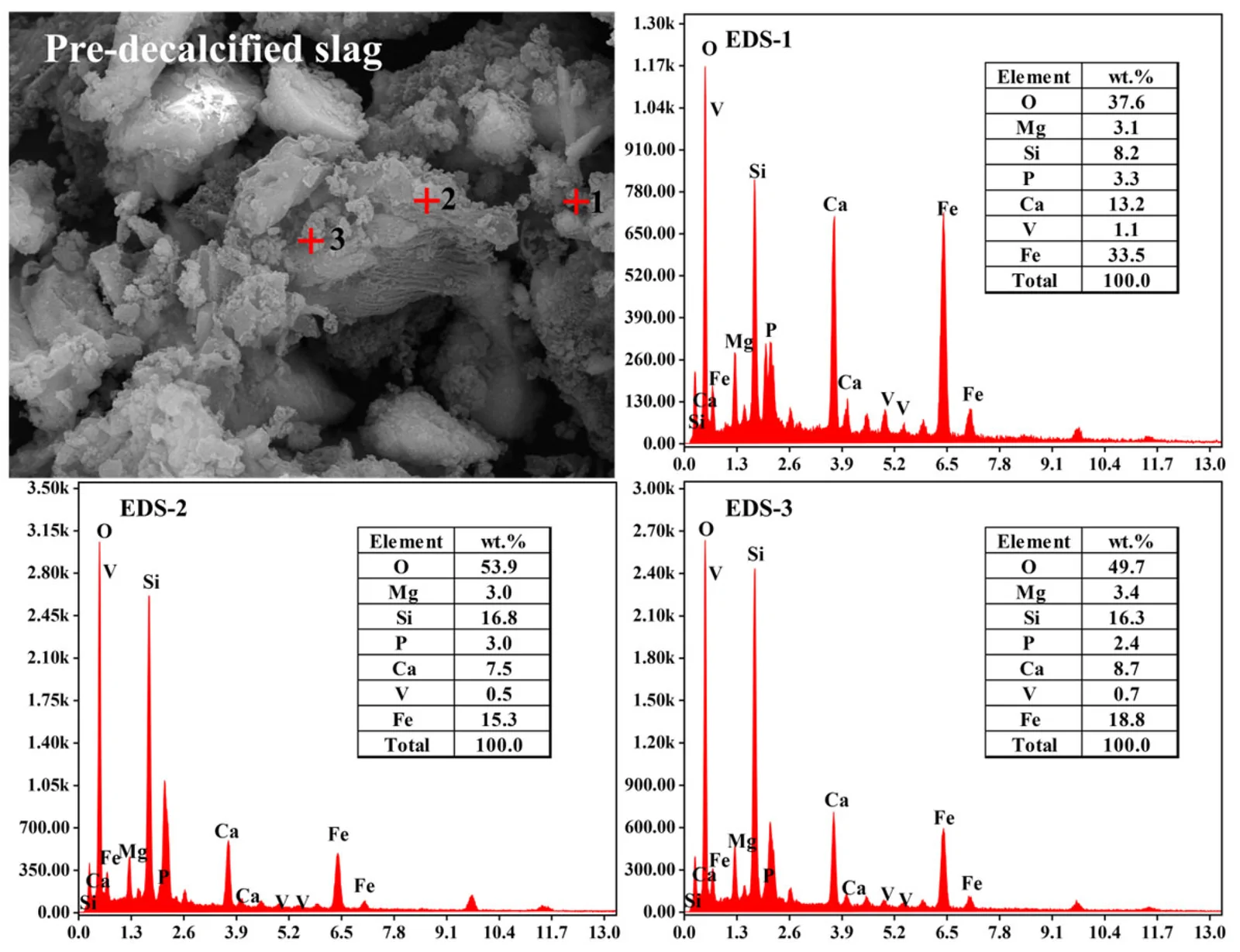
SEM image and EDS point analysis of pre-decalcified slag [25].

To further identify the composition of these regions, EDS point analyses were conducted (Figure 13) and interpreted together with the XRD results. The three representative points are mainly composed of Fe and Si, accompanied by small amounts of residual Ca, indicating the presence of Fe-enriched phases and calcium-depleted silicate frameworks.

Overall, the decalcification process significantly alters both the microstructure and elemental distribution of the slag. The original Ca-Fe-Si multiphase intergrowth structure is transformed into a heterogeneous residue dominated by Fe-enriched phases and silicate remnants. The preferential dissolution of calcium-rich phases enables the selective leaching of Ca, whereas vanadium remains relatively stable because it is primarily incorporated into the residual solid phases through isomorphous substitution. These observations provide microstructural evidence that differences in phase stability are instrumental in governing the selective leaching behavior of elements in selective decalcification.

**Figure 14:**
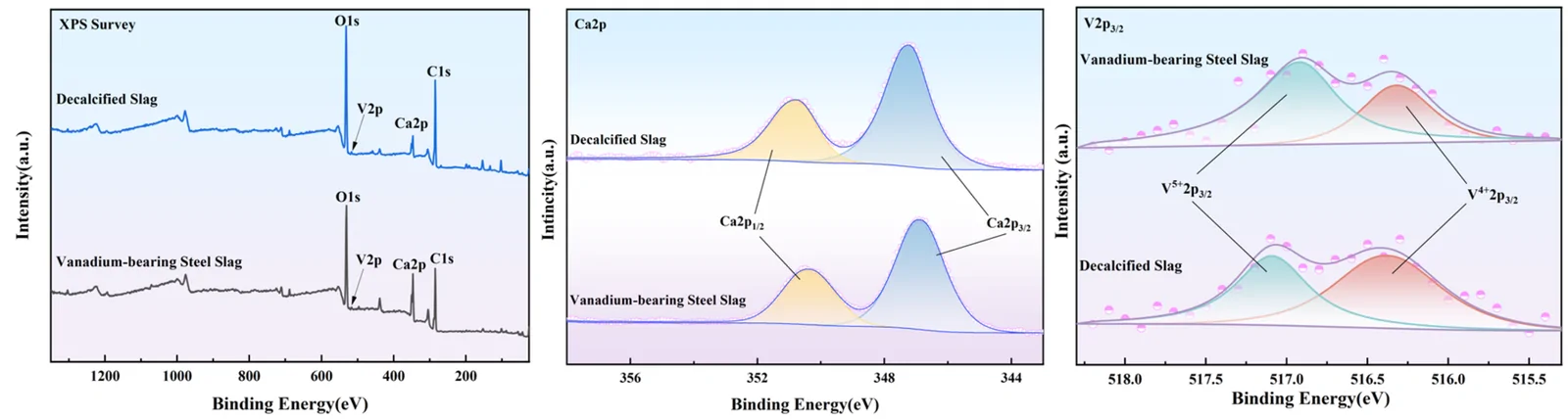
XPS spectra of vanadium-bearing steel slag and pre-decalcified slag.

The XPS results further confirm the substantial changes in surface chemical composition induced by selective decalcification, as shown in Figure 14. The survey spectrum displays a marked attenuation of the Ca signal after leaching, whereas the relative intensity of the V signal becomes stronger, indicating the effective removal of calcium-bearing phases and the relative enrichment of vanadium.

The Ca 2p spectrum exhibits a typical spin–orbit doublet with components at 347.2 eV (Ca 2p_3/2_) and 350.5 eV (Ca 2p_1/2_). The peak area ratio is approximately 2:1, which is characteristic of Ca^2+^ in silicate and ferrite lattices [26]. These binding energies correspond to the Ca-O bonding environments in C_2_S and C_2_F. In vanadium-bearing steel slag, calcium predominantly exists in the form of Ca-O ionic bonds. Although these bonds exhibit strong ionic character, their bond strength is relatively low. According to the bond energy database of the Department of Chemistry, University of California, Santa Barbara, the bond energy of Ca-O is 464 ± 84 kJ·mol^−1^ [27]. Under acidic conditions, H^+^ readily reacts with lattice O^2−^ to form H_2_O, thereby weakening and cleaving Ca-O bonds and facilitating the release of Ca^2+^ into solution. Consequently, calcium exhibits high leaching reactivity and becomes the preferentially dissolved component during the decalcification process.

Vanadium in the slag is mainly associated with C_2_S and C_2_F phases in the form of V^5+^ and V^4+^. A small fraction of V^5+^ occupies Si sites in tetrahedral coordination, whereas the majority of V^5+^ is enriched in the boundary regions of the C_2_S phase. In contrast, V^4+^ preferentially substitutes for Fe^3+^ at octahedral sites within the ferrite structure [28].

The V 2p spectrum shows a V 2p_3/2_ peak at 517.1 eV, which is indicative of V^5+^ in the raw slag [29]. Owing to the relatively low vanadium content, the V2p_1/2_ signal is obscured by background noise, and therefore only the V2p_3/2_ region is fitted and analyzed. In addition, a weaker component at 516.4 eV is identified and attributed to V^4+^ substituting for Fe^3+^ at octahedral sites in the calcium ferrite phase.

Unlike Ca-O bonds, V-O bonds possess a pronounced covalent character, particularly within VO_4_ tetrahedral and VO_6_ octahedral coordination structures. The bond energy of V-O is approximately 644 kJ·mol^−1^ [30], which substantially exceeds that of Ca-O bonds. Furthermore, the multiple coordination environments surrounding vanadium provide greater local structural stability, making V-O bonds less susceptible to cleavage under the relatively mild acidic conditions employed in this study. The dispersed occurrence of vanadium within the mineral lattice further enhances its resistance to dissolution. Although a fraction of vanadium can be liberated upon dissolution of readily soluble phases such as C_2_S, a considerable proportion remains incorporated within the more stable ferrite phases and residual silicate frameworks. As a result, vanadium exhibits an overall retention behavior during decalcification. Therefore, from the perspectives of bond energy and coordination chemistry, the lower stability of Ca-O bonds and the greater resistance of V-O bonds to acid attack constitute the fundamental mechanism responsible for selective calcium removal with vanadium retention.

This interpretation is consistent with the disappearance of C_2_S observed by XRD, the retention of C_2_F in the solid residue, and the slight increase in V_2_O_5_ content from 2.19 wt.% to 2.49 wt.% determined by ICP analysis. Collectively, these results confirm the selective removal of calcium-bearing phases and the effective enrichment of vanadium during the decalcification process.

The above characterization results demonstrate that the combined mechanical activation–hydrochloric acid pretreatment enables the selective decalcification of vanadium-bearing steel slag. After decalcification, only C_2_F and FeO remain as the dominant phases, whereas calcium-bearing phases such as C_2_S are largely eliminated or they are transformed into amorphous phases. Vanadium is predominantly retained in the solid residue as V^5+^ and V^4+^, primarily associated with the C_2_F phases and residual silicate frameworks. Therefore, the resulting pre-decalcified slag can serve as an intermediate feedstock for subsequent vanadium extraction and recovery.

### 2.4. Kinetic Analysis

The decalcification of vanadium-bearing steel slag in hydrochloric acid belongs to the category of liquid–solid heterogeneous reactions. During the decalcification process, the selective dissolution of calcium-bearing phases leads to chemical reconstruction of the particle surface. However, vanadium-bearing C_2_F, residual silicate phases, and FeO are retained as a solid skeleton. The persistence of this framework maintains the overall particle size during leaching, thereby satisfying the fundamental assumption of the shrinking-core model assuming invariant particle dimensions. This result corroborates the discussion by Liddell et al. [31] regarding the applicability of shrinking-core models in hydrometallurgical systems, where the constant-particle-size model is considered more appropriate than the shrinking-particle model when an inert framework or solid product layer supports the particle structure during liquid–solid reactions. To elucidate the kinetic behavior of calcium dissolution during decalcification, the shrinking-core model assuming invariant particle dimensions was employed to investigate how hydrochloric acid concentration and reaction temperature affect the leaching kinetics. According to the characteristics of liquid–solid heterogeneous reactions, three kinetic models were considered, including diffusion control through the solid product layer, chemically controlled surface reactions, and mixed control, for discerning the rate-limiting mechanism.

When the decalcification process is limited by internal diffusion, the kinetic equation takes the form of Equation (1):

(1) $$1-\left(2/3\right)x-{\left(1-x\right)}^{2/3}=k_{d}t,$$

When the process is controlled by surface chemical reaction, the kinetic equation can be expressed as Equation (2):

(2) $$1-{\left(1-x\right)}^{1/3}=k_{d}t,$$

When the process is governed by mixed control, the kinetic equation can be expressed as Equation (3):

(3) $${\left(1-x\right)}^{-1/3}-1+1/3\ln\left(1-x\right)=k_{d}t,$$

where

*x*—the Ca leaching rate (%),
*t*—the reaction time (min),
*k_d_*—the apparent rate constant corresponding to the respective controlling mechanism (min^−1^).

The temperature dependence of the apparent rate constant (*k_d_*) follows the Arrhenius equation:

(4) $$k_{d}=A\exp\left(-\frac{E_{a}}{RT}\right),$$

Logarithmic transformation of both sides produces

(5) $$\ln K_{d}=-\frac{E_{a}}{RT}+\ln A,$$

where:

*E_a_*—the apparent activation energy (kJ·mol^−1^),
*R*—the universal gas constant (8.314 J·mol^−1^·K^−1^),
*T*—the absolute temperature (K),
*A*—the pre-exponential factor (s^−1^).

#### 2.4.1. Effect of HCl Concentration on Decalcification Rate

**Figure 15:**
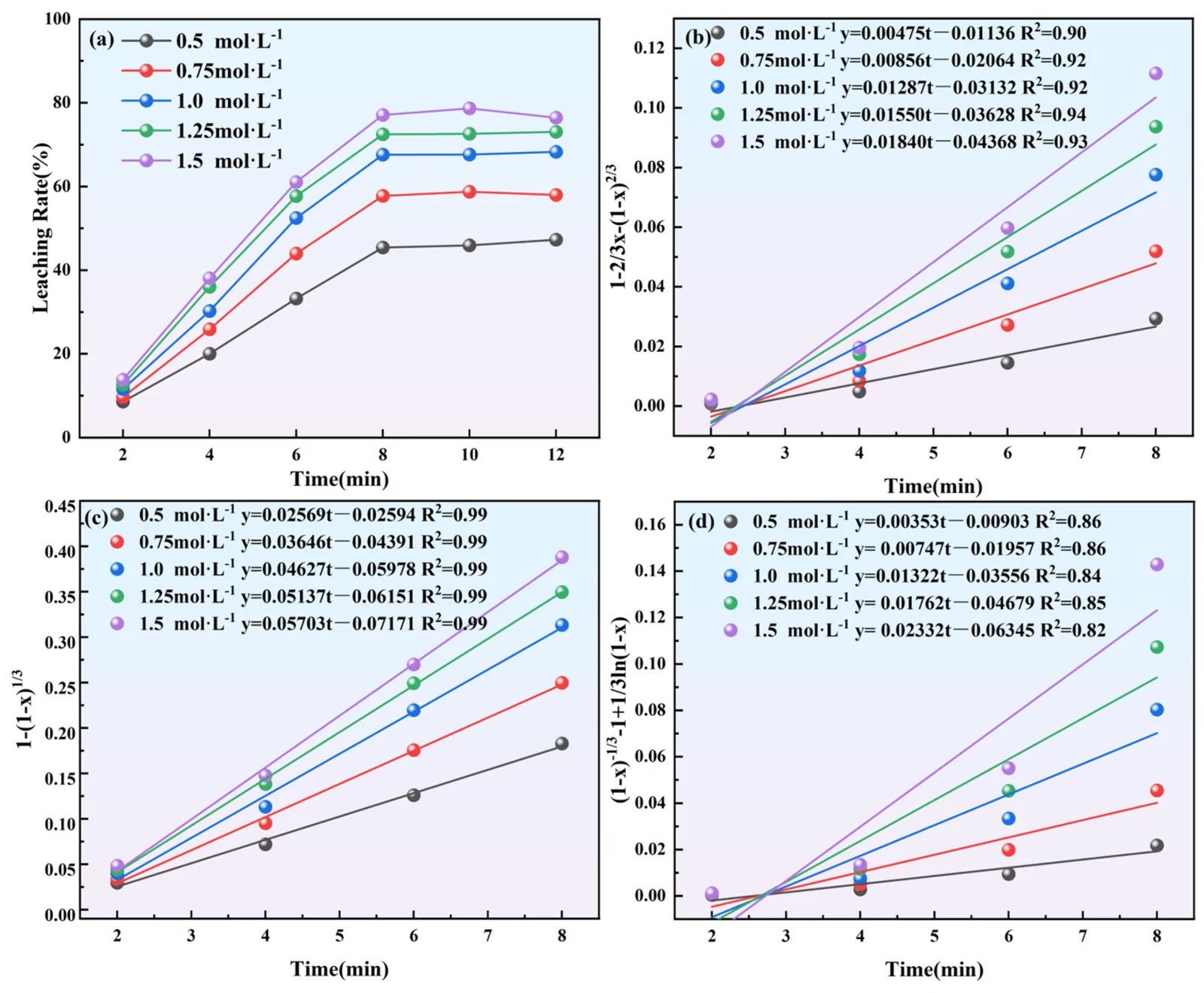
Kinetic fitting curves of the decalcification process at different HCl concentrations: (**a**) effect of HCl concentration on the decalcification process; (**b**) linear relationship between 1 − 2/3*x* − (1 − *x*)^2/3^ and *t*; (**c**) linear relationship between 1 − (1 − *x*)^1/3^ and *t*; (**d**) linear relationship between (1 − *x*)^−1/3^ − 1 + 1/3ln(1 − *x*) and *t*.

Under fixed conditions of an L/S ratio of 7 mL·g^−1^, 8 min, and 40 °C, the effect of hydrochloric acid concentration (0.5–1.5 mol·L^−1^, spaced by 0.25 mol·L^−1^) on the decalcification kinetics was investigated. Figure 15a shows the calcium leaching behavior at different hydrochloric acid concentrations. During the initial stage (0–8 min), the Ca leaching rate increased markedly with increasing hydrochloric acid concentration and exhibited an approximately linear dependence on acid concentration. Beyond 8 min, the leaching rate gradually approached a plateau with no further significant increase, indicating that calcium dissolution had essentially reached equilibrium. Therefore, only the data obtained within the first 8 min were used for kinetic fitting to identify the rate-limiting step, as illustrated in Figure 15b–d.

Among the three kinetic models examined, the surface chemical reaction-controlled model (Figure 15c) exhibited the highest coefficient of determination (R^2^) and the smallest deviation between the theoretical predictions and the experimental data. In contrast, the internal diffusion-controlled model (Figure 15b) showed a slightly poorer fit throughout the reaction period. The mixed-control model (Figure 15d) exhibited the largest fitting deviation, indicating inadequate predictive accuracy.

**Figure 16:**
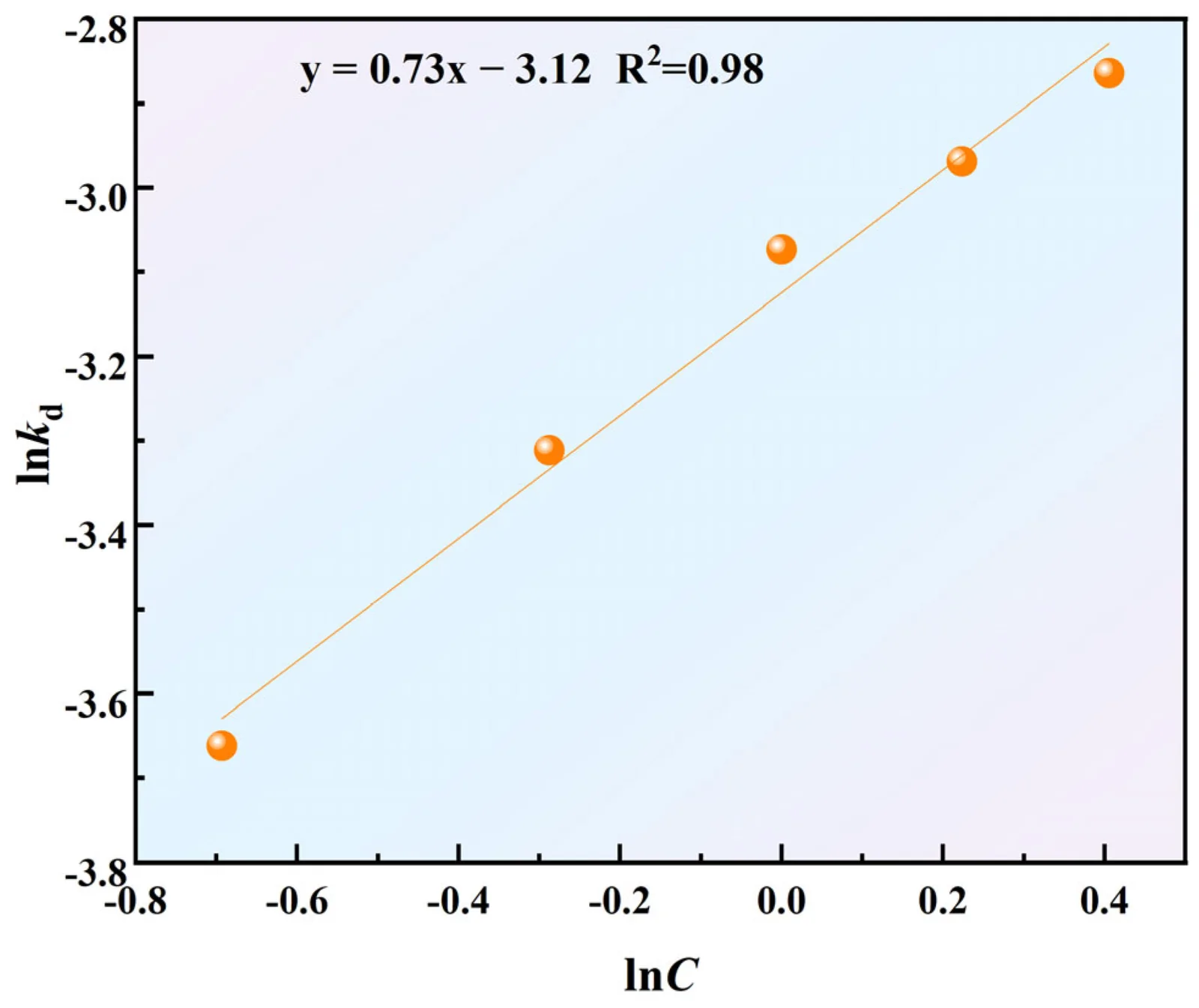
Relationship between the rate constant and the logarithm of HCl concentration.

The kinetic fitting results demonstrated that the selective decalcification process followed a shrinking-core model controlled predominantly by interfacial chemical kinetics. To further quantify how hydrochloric acid concentration affects calcium dissolution, the apparent rate constants (*k*_d_) were derived by linear regression of the kinetic data presented in Figure 15c. The relationship between ln *k*_d_ and ln *C* is presented in Figure 16. The slope of the fitted line was 0.73, with a standard error of 0.05 based on linear regression. Corresponding to an apparent reaction order of *n* = 0.73 with respect to HCl concentration.

This fractional reaction order is consistent with the surface chemical reaction-controlled behavior identified by the kinetic model fitting [32]. The non-integer dependence on acid concentration may be associated with the heterogeneous distribution of reactive sites in the multiphase vanadium-bearing steel slag.

#### 2.4.2. Effect of Temperature on Decalcification Rate

**Figure 17:**
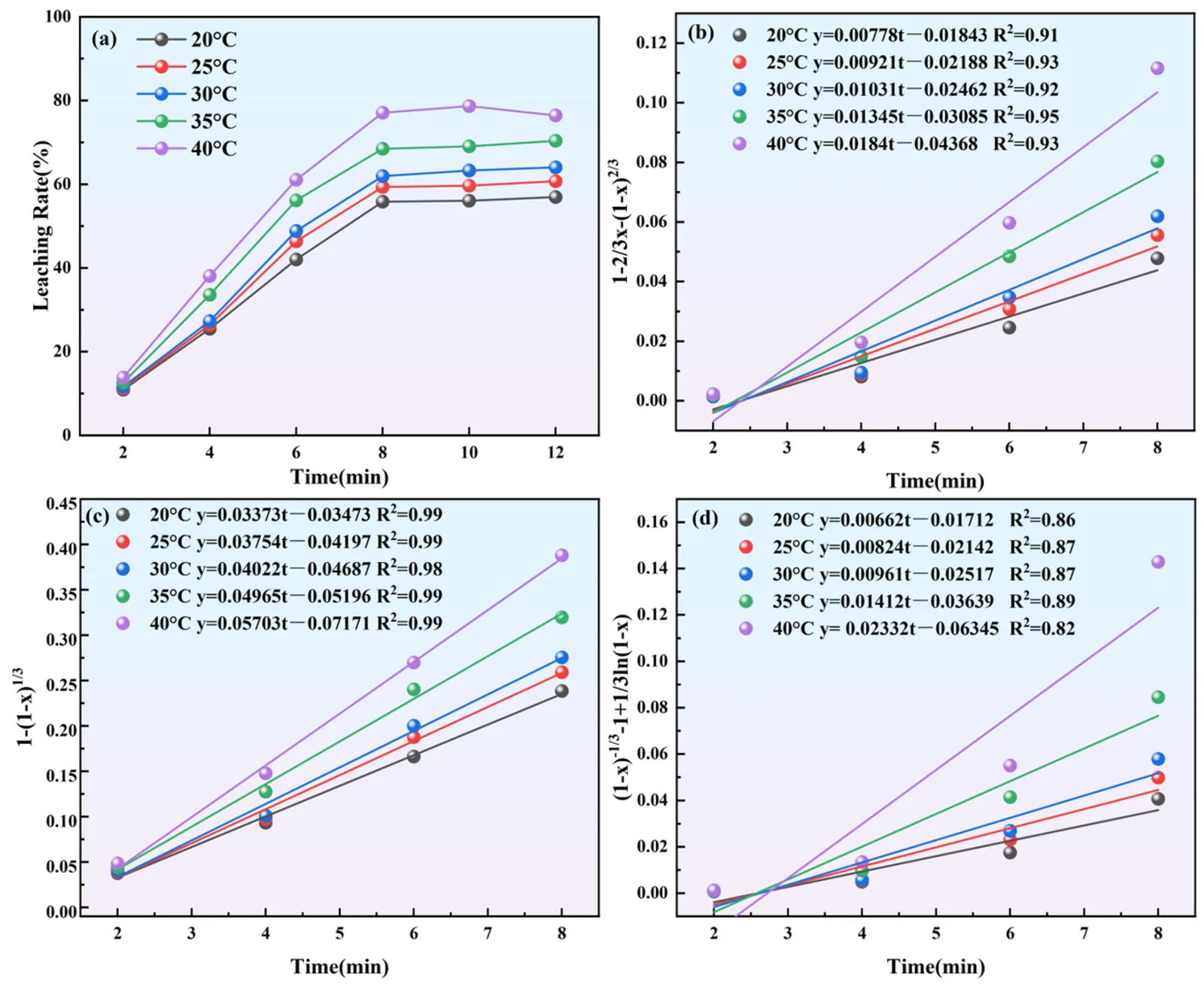
Kinetic fitting curves of the decalcification process at different temperatures: (**a**) effect of temperatures on the decalcification process; (**b**) linear relationship between 1 − 2/3*x* − (1 − *x*)^2/3^ and *t*; (**c**) linear relationship between 1 − (1 − *x*)^1/3^ and *t*; (**d**) linear relationship between (1 − *x*)^−1/3^ − 1 + 1/3ln(1 − *x*) and *t*.

Under fixed conditions of 1.5 mol·L^−1^ HCl, 8 min, and an L/S ratio of 7 m·g^−1^, the influence of temperature (20–40 °C, with an interval of 5 °C) on the decalcification kinetics was investigated. The calcium leaching behavior at different temperatures is presented in Figure 17a. During the initial reaction stage (0–8 min), Ca leaching was considerably promoted by increasing temperature and exhibited an approximately linear dependence on reaction time. Beyond 8 min, the Ca leaching rate gradually approached a plateau, indicating that calcium dissolution had essentially reached equilibrium. Therefore, only the data obtained within the first 8 min were selected for kinetic fitting to identify the rate-controlling step.

As illustrated in Figure 17b–d, the fitting outcomes demonstrated that the surface chemical reaction-controlled model (Equation (2)) showed the most satisfactory consistency with the experimental data at all investigated temperatures, reflected in the highest correlation coefficients (R^2^) and the smallest fitting deviations among the three kinetic models. These results indicate that the dissolution of calcium was described by a shrinking-core mechanism with chemical reactions at the solid–liquid interface serving as the dominant rate-controlling step within 20–40 °C.

**Figure 18:**
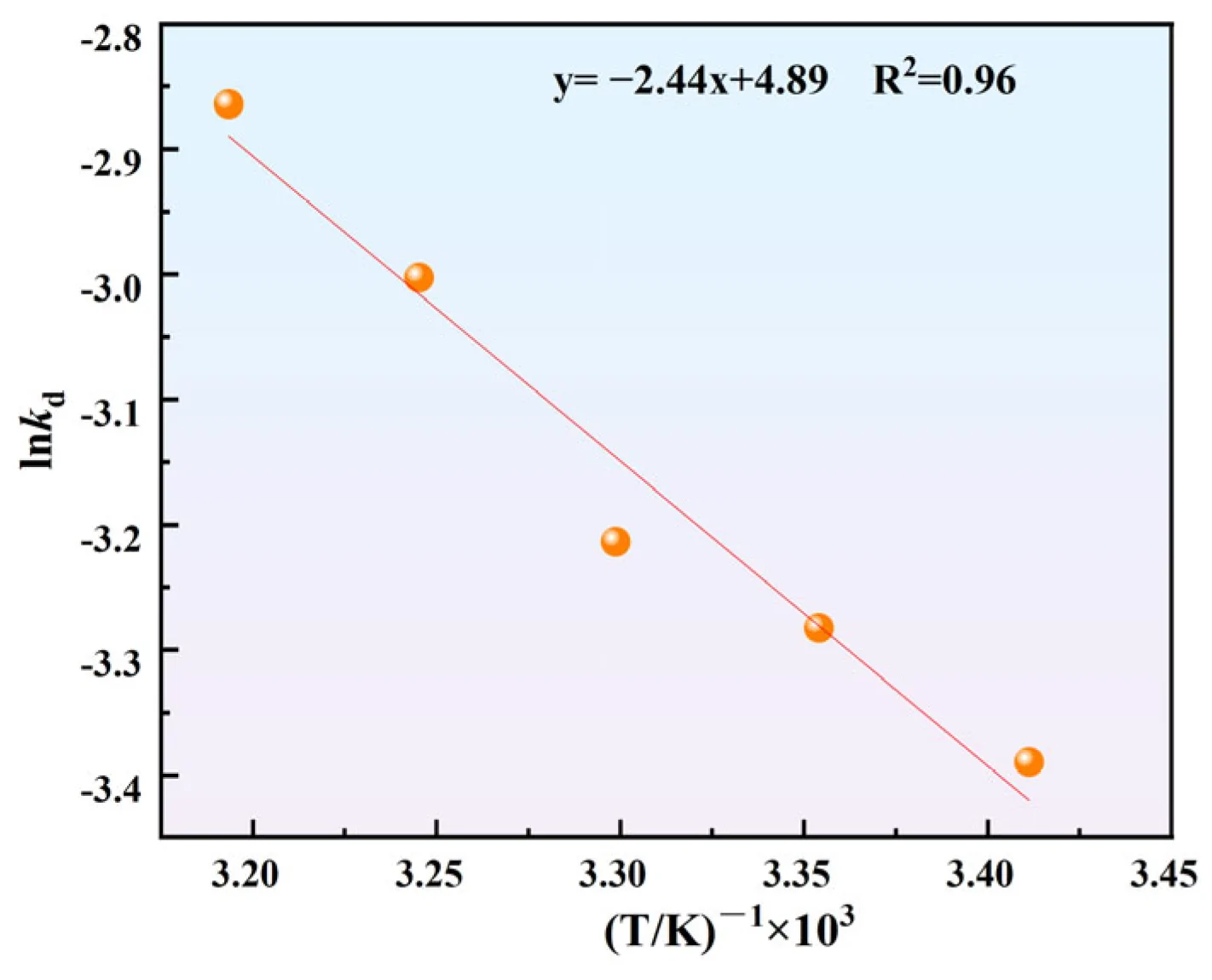
Arrhenius plot of ln *k*_d_ versus 1000/*T* for the decalcification process.

To further evaluate the temperature dependence of the decalcification process, the apparent rate constants obtained from the surface chemical reaction-controlled model were fitted using the Arrhenius equation, and the corresponding results are presented in Figure 18. The apparent activation energy was determined to be 20.28 kJ·mol^−1^, with a standard error of 2.21 kJ·mol^−1^ derived from the linear regression.

For mechanically activated systems containing porous residual layers, the apparent activation energy may be substantially reduced while decalcification remains controlled by surface chemical reaction. Liu et al. [33] reported an apparent activation energy of 20.87 kJ·mol^−1^ for the pressure acid leaching of vanadium-bearing steel slag and identified the process as being governed by surface chemical reaction. In addition, Li et al. [23] showed that mechanical activation markedly enhances the reactivity of steel slag through lattice distortion and defect generation, which is consistent with the reduction in apparent activation energy observed in mechanically activated systems. Combined with the superior fitting performance of the surface chemical reaction-controlled model, the apparent activation energy of 20.28 kJ·mol^−1^ observed in the present study can be reasonably interpreted as reflecting the enhanced reactivity induced by mechanical activation rather than excluding a chemically dominated process. Therefore, interfacial chemical reaction was considered the predominant rate-controlling step for decalcification.

## 3. Materials and Methods

### 3.1. Materials

The vanadium-bearing steel slag used in this study was obtained from a steel plant in Chengde, Hebei Province, China. After grinding, the raw slag exhibited a D10–D90 particle-size range of 10.542–490.765 μm with a median particle size (D50) of 146.870 μm. Following complete digestion using a mixed acid solution of aqua regia and hydrofluoric acid at a volume ratio of 9:1, the major elements were quantified via inductively coupled plasma optical emission spectrometry (ICP-OES, 725 ES, Agilent Technologies, Mulgrave, Australia). External calibration was performed using certified standard solutions prior to sample analysis. The results are presented in Table 2. CaO is the principal constituent (40.83 wt.%), while the vanadium content, expressed as V_2_O_5_, is 2.19 wt.%, indicating that the material is a high-calcium, low-vanadium slag. Despite its relatively low vanadium content, the slag contains more vanadium than stone coal (0.3–1.0 wt.% V_2_O_5_), underscoring its viability as a secondary vanadium resource.

**Figure 19:**
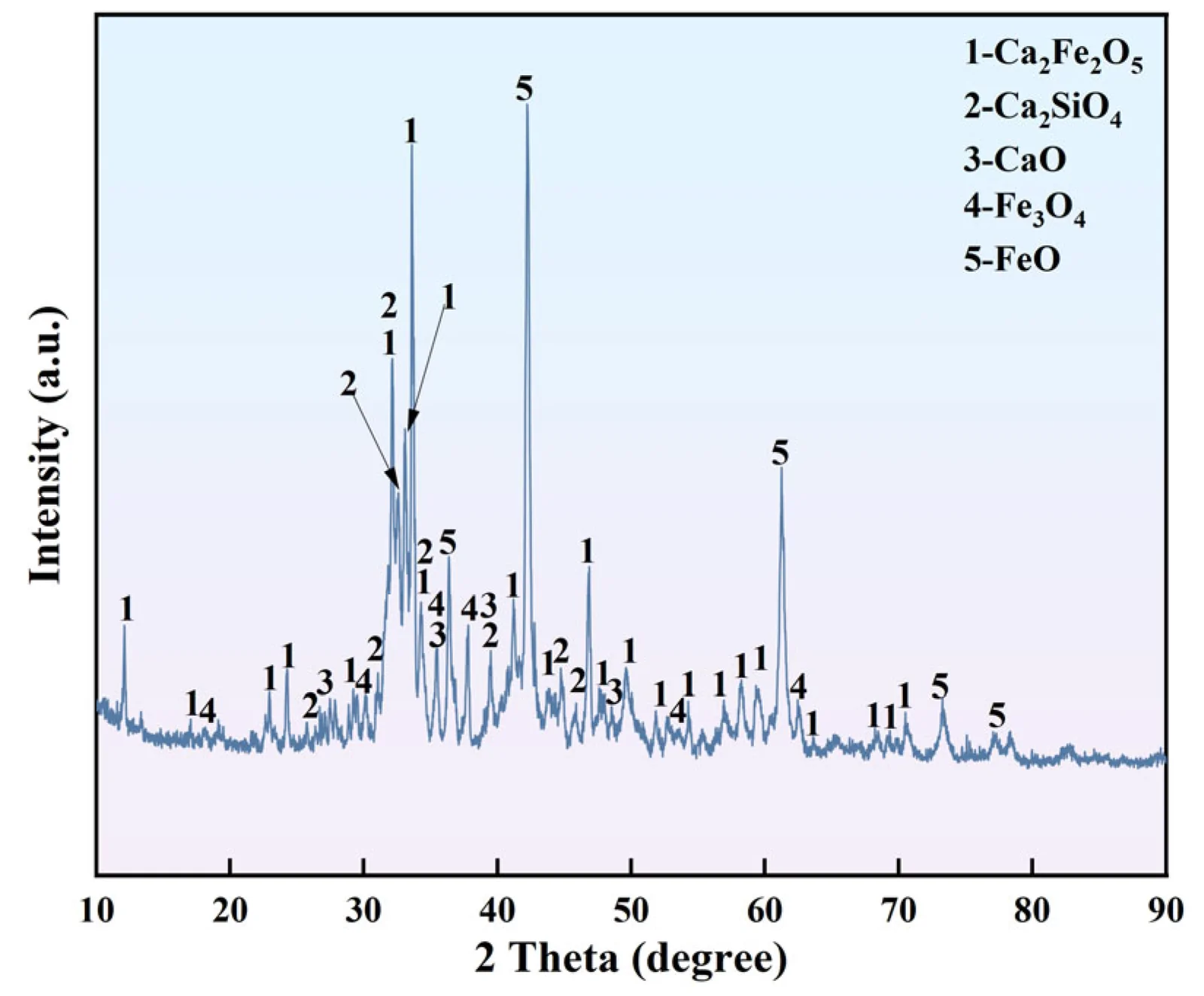
XRD pattern of vanadium-bearing steel slag.

The mineralogical composition of this vanadium-bearing steel slag was examined using X-ray diffraction (XRD, D/MAX2500PC, Rigaku Corporation, Akishima, Japan), using Cu Kα radiation at 40 kV and 40 mA over a 2θ range of 10–90° with a scanning rate of 0.12°·s^−1^, and the diffraction pattern is presented in Figure 19. The slag was mainly composed of dicalcium ferrite (C_2_F), dicalcium silicate (C_2_S), and RO phases, whereas no discrete crystalline V_2_O_5_ phase was detected.

**Figure 20:**
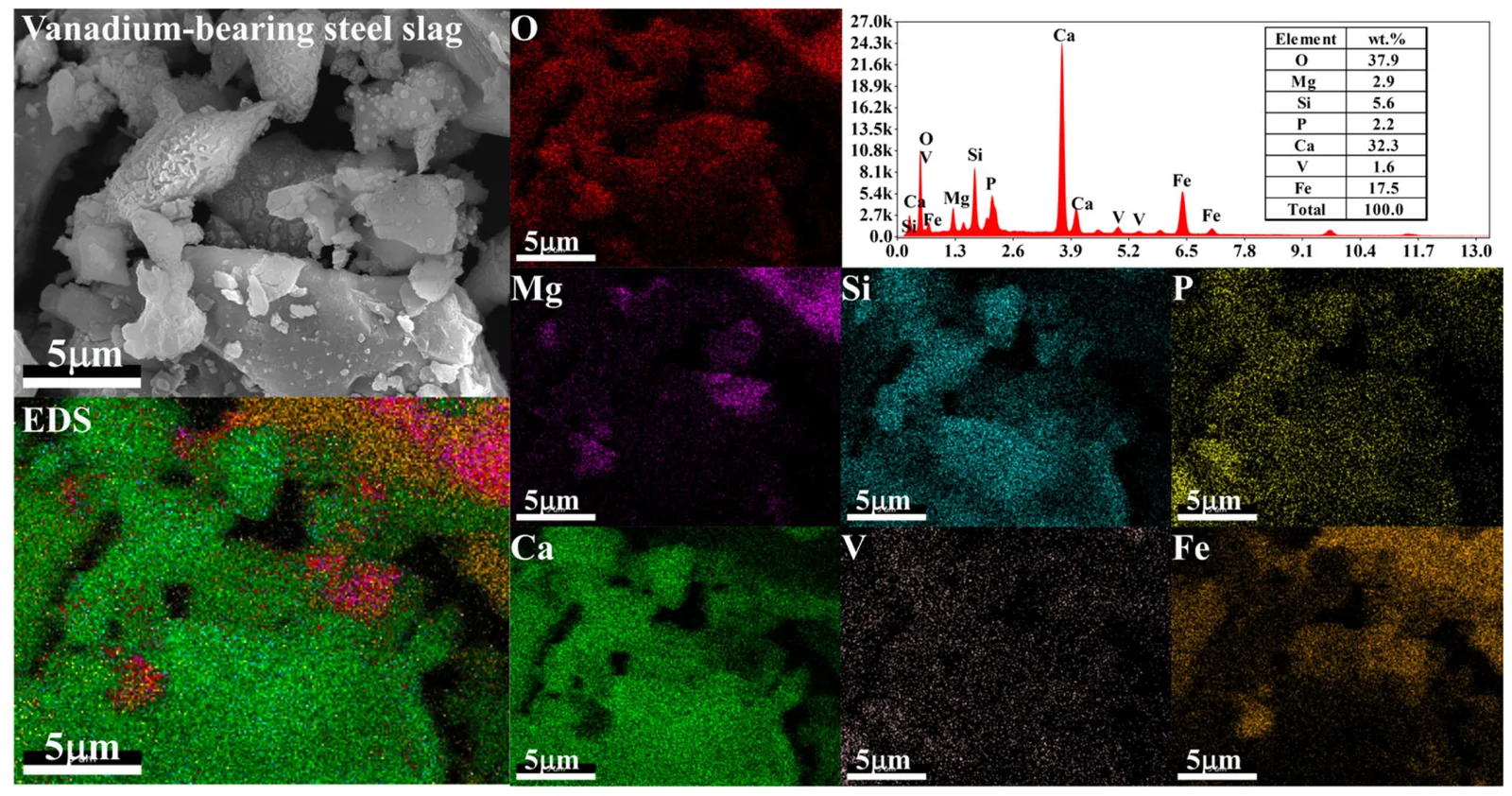
SEM image and EDS elemental mapping of vanadium-bearing steel slag.

To further elucidate the occurrence state of vanadium, scanning electron microscopy coupled with energy-dispersive spectroscopy (SEM-EDS, Quattro S, Thermo Fisher Scientific, Hillsboro, OR, USA, equipped with an EDAX Elect Plus EDS detector, AMETEK, Mahwah, NJ, USA) was performed to examine the slag microstructure and map the distribution of constituent elements. SEM observations were conducted under high-vacuum conditions with a working distance of 8.2 mm and a spot size of 2.0, using secondary-electron imaging with an ETD detector. The SEM image and corresponding elemental mapping results are shown in Figure 20. The slag exhibited a dense and compact morphology. Elemental mapping revealed two major enrichment regions: a Ca-Fe co-enriched region located in the upper-right area and a Ca-Si co-enriched region in the lower-left area. Combined with the XRD results, the former was attributed to the C_2_F phase together with part of the RO phase, whereas the latter mainly corresponded to the C_2_S phase.

Vanadium showed a relatively dispersed distribution throughout the slag matrix. According to EPMA results reported for similar slag systems [34], C_2_S and C_2_F are the principal vanadium-bearing phases in vanadium-bearing steel slag. Although the vanadium content in C_2_S is lower than that in C_2_F, the substantially higher abundance of C_2_S makes it the dominant host phase for vanadium, whereas the RO phase contains only a minor amount of vanadium. Therefore, vanadium is inferred to occur mainly through isomorphic substitution within calcium-bearing phases such as dicalcium ferrite and dicalcium silicate.

**Figure 21:**
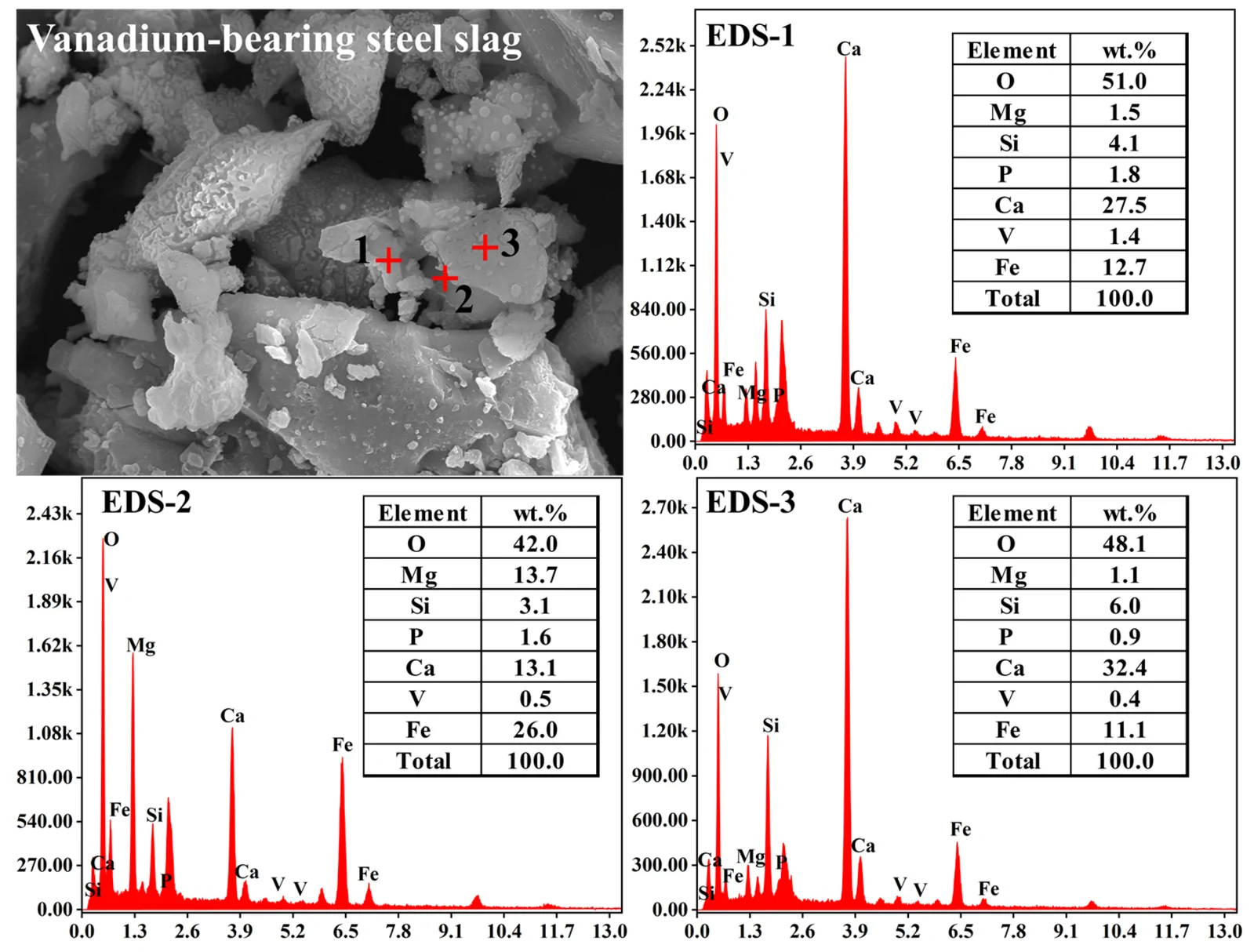
SEM image and EDS point analysis of vanadium-bearing steel slag.

The elemental mapping results further indicate that the slag is not a homogeneous single-phase material but rather a heterogeneous Ca-Fe-Si multiphase assemblage with intricate phase intergrowths. Representative EDS point analyses are shown in Figure 21. Point 1 corresponds to a multiphase region without a clearly dominant phase, which is likely associated with the intergrowth of silicate and Fe-rich phases. Point 2 is characterized by the coexistence of Ca and Fe, with Fe being the dominant element. Combined with the characteristic diffraction peaks of C_2_F and iron oxides identified by XRD, this region is considered to be associated with ferrite phases. Point 3 is enriched in Ca and Si and is therefore attributed primarily to silicate phases.

**Figure 22:**
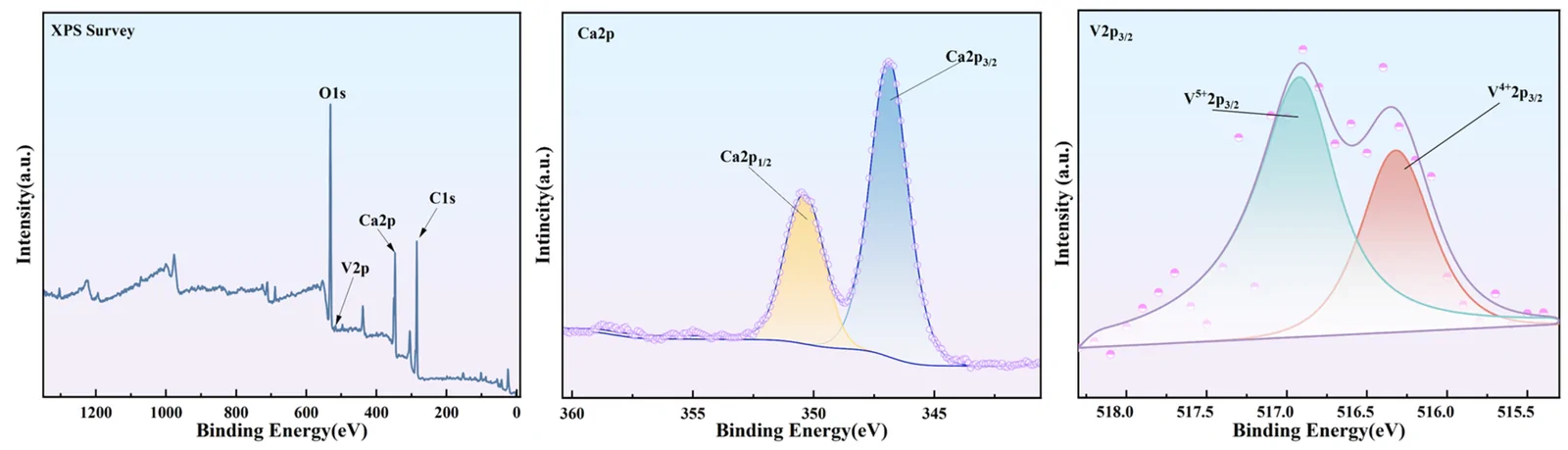
XPS spectra of vanadium-bearing steel slag.

The chemical states of Ca and V in the vanadium-bearing steel slag were investigated by X-ray photoelectron spectroscopy (XPS, K-Alpha, Thermo Fisher Scientific, Hillsboro, OR, USA), with the resulting spectra shown in Figure 22. The survey spectrum indicates that the slag surface is mainly composed of C, O, Ca, and V. The C 1s peak at 284.8 eV originates from adventitious carbon and was used for charge correction. The XPS spectra were processed and peak-fitted using Thermo Avantage software 5.9. A Smart background was applied, and peak deconvolution was performed using mixed Gaussian–Lorentzian line shapes.

The Ca 2p spectrum exhibits a typical spin–orbit doublet with components at 346.8 eV (Ca 2p_3/2_) and 350.4 eV (Ca 2p_1/2_). The peak area ratio is approximately 2:1, which is characteristic of Ca^2+^ in silicate and ferrite lattices.

The V 2p spectrum shows a V 2p_3/2_ peak at 516.9 eV and 516.3 eV, which is indicative of V^5+^ and V^4+^. Owing to the relatively low vanadium content, the V2p_1/2_ signal is obscured by background noise, and therefore only the V2p_3/2_ region is fitted and analyzed.

### 3.2. Experimental Methods

In this study, “vanadium-bearing steel slag” refers to the original slag before hydrochloric acid decalcification, whereas “pre-decalcified slag” refers to the solid residue obtained after hydrochloric acid decalcification. Mechanical activation of the vanadium-bearing steel slag was carried out using a planetary ball mill (XQM-2); 304 stainless steel grinding balls (10 and 5 mm in diameter) were mixed at a mass ratio of 1:1, and the ball-to-powder ratio was maintained at 15:1. During milling, 3 wt.% anhydrous ethanol (analytical grade, Shanghai Macklin Biochemical Co., Ltd., Shanghai, China) was added as a process control agent to minimize particle agglomeration. The milling speed was set at 350 r·min^−1^. To prevent excessive heat accumulation, the mill was operated in an alternating rotational mode with a cycle of 15 min milling followed by a 5 min pause. The total milling time was 30 min. After mechanical activation, the slag exhibited a D10–D90 particle-size range of 11.713–383.027 μm, with a median particle size (D50) of 119.426 μm.

After milling, the milled products were allowed to cool to room temperature inside the milling jar, and subsequently separated from the grinding media using a 304 stainless-steel standard sieve. The activated samples were dried and immediately used for subsequent decalcification experiments to minimize the influence of surface alterations caused by environmental exposure. In this study, mechanical activation served solely as a pretreatment step to enhance slag reactivity and ensure sample consistency throughout the experiments. To avoid introducing additional variables, all milling parameters were kept constant during the entire study.

For the decalcification experiments, 5 g of either activated or non-activated vanadium-bearing steel slag was mixed with hydrochloric acid (Sinopharm Chemical Reagent Co., Ltd., Shanghai, China) solutions of varying concentrations (0.5–2.5 mol·L^−1^) at different L/S ratios (3–11 mL·g^−1^) in clean beakers. The mixtures were subjected to thermostatic water bath treatment at 20–60 °C with continuous stirring at 600 r·min^−1^ for 5–17 min. After the reaction, solid–liquid separation was carried out by vacuum filtration, yielding a leachate and a solid residue. The solid residue was rinsed twice with distilled water and subsequently dried in an oven before further characterization.

**Figure 23:**
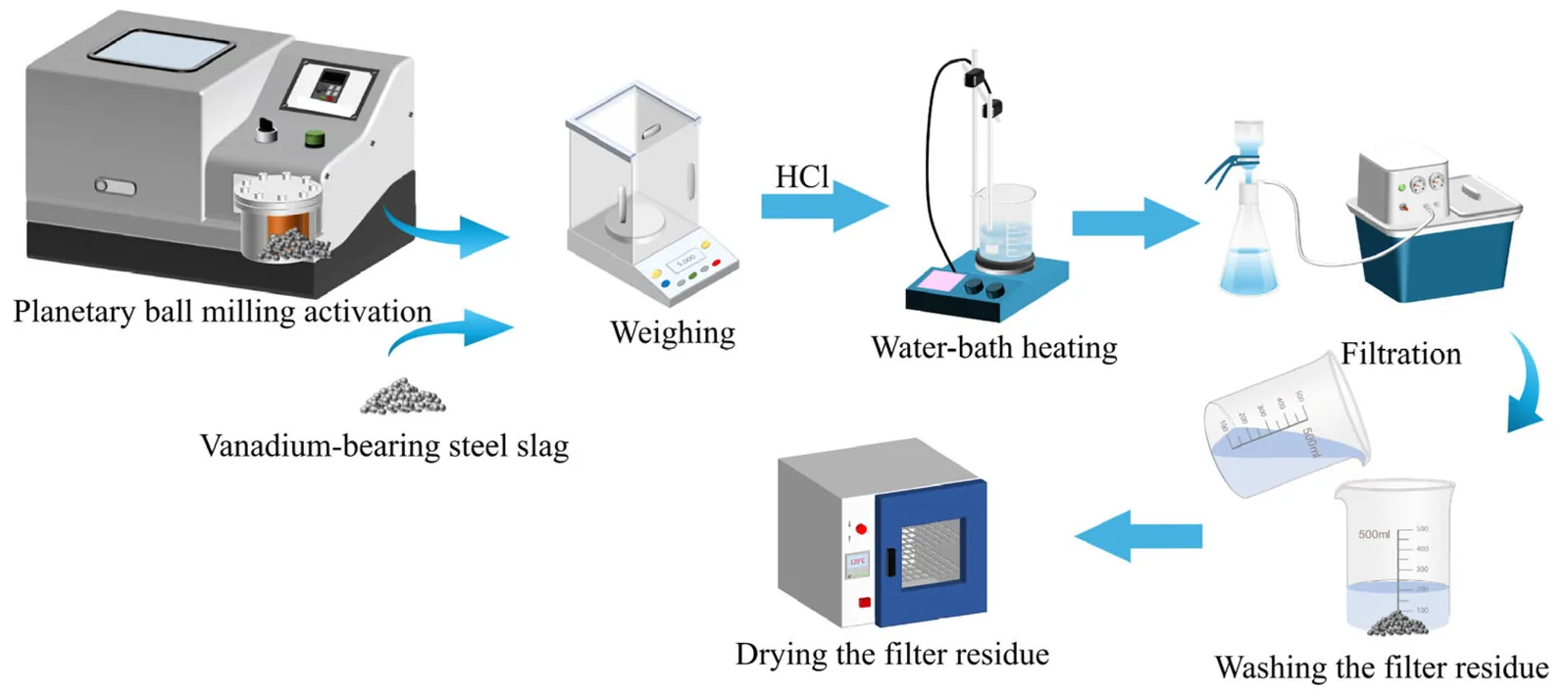
Schematic diagram of the experimental procedure.

The concentrations of dissolved elements in the leachates were determined by ICP-OES, and the corresponding leaching rates were determined based on Equation (6). Figure 23 presents a schematic diagram of the experimental procedure.

(6) $$E_{i}=\frac{c_{i}\times \;V}{m\times w_{i}}\times 100\%,$$

where

*E_i_*—extraction rate of element *i* (%);
*c_i_*—measured concentration of element *i* in solution (g·L^−1^);
*V*—bulk volume of the leaching solution (L);
*m*—initial mass of the solid specimen (g);
*w_i_*—the mass fraction of element *i* in the raw solid (dimensionless).

To quantitatively evaluate the selectivity of the decalcification process and maximize calcium removal while minimizing vanadium loss, a Separation Index (*SI*) is defined as the ratio of the calcium leaching rate to the vanadium leaching rate, as shown in Equation (7). A higher *SI* value indicates more effective calcium removal with lower relative vanadium loss and is therefore more favorable for vanadium enrichment in the residue.

(7) $$SI_{\frac{\mathrm{Ca}}{V}}=\frac{E_{\mathrm{Ca}}}{E_{V}},$$

where

*SI*_Ca/V_—the Separation Index, defined as Ca leaching rate divided by V leaching rate (dimensionless);
*E*_Ca_—the Ca leaching rate (%);
*E*_V_—the V leaching rate (%).

## 4. Conclusions

1. The influence of HCl concentration, reaction temperature, reaction time, and liquid-to-solid ratio on the leaching behaviors of Ca and V from mechanically activated and non-activated vanadium-bearing steel slag were systematically investigated. For mechanically activated slag, under the optimal conditions of 1.5 mol·L^−1^ HCl, 40 °C, 8 min, and a liquid-to-solid ratio of 7 mL·g^−1^, the Ca leaching rate reached 77.10%, while the V loss rate remained as low as 6.08%, yielding a maximum Separation Index of 12.7.
2. XRD and SEM-EDS characterization revealed that the vanadium-bearing steel slag chiefly contained dicalcium ferrite (C_2_F), dicalcium silicate (C_2_S), CaO, Fe_3_O_4_, and FeO phases. Vanadium was primarily incorporated into calcium-bearing phases through isomorphous substitution. After decalcification, only C_2_F and FeO were detected, while the compact and smooth morphology of the raw slag transformed into a loose structure with evident dissolution features, indicating substantial destruction of the original mineral structure. ICP analysis revealed that the CaO content decreased from 40.83 wt.% to 19.87 wt.%, whereas the V_2_O_5_ content increased from 2.19 wt.% to 2.49 wt.%. These results demonstrated the preferential dissolution of calcium-bearing phases and the consequent enrichment of vanadium in the solid residue. The decalcification process therefore exhibited pronounced structural selectivity, providing favorable conditions for subsequent vanadium extraction.
3. During the initial 8 min of reaction, calcium dissolution followed a shrinking-core model with invariant particle dimensions, and the overall kinetics was governed by interfacial chemical processes. Arrhenius analysis yielded an apparent activation energy of 20.28 kJ·mol^−1^. The apparent reaction order with respect to hydrochloric acid concentration was calculated to be 0.73. Accordingly, the semi-empirical kinetic expression takes the following form: ln*k_d_* = 0.73ln*C* − 3.12, where *k_d_* is the apparent rate constant and *C* is the hydrochloric acid concentration.

## Figures and Tables

**Table 1:** Main chemical compositions of vanadium-bearing steel slag and pre-decalcified slag (mass fraction, %).

Chemical Composition	CaO	Fe_2_O_3_	MgO	V_2_O_5_	P_2_O_5_	SiO_2_
Vanadium-bearing steel slag	40.83	30.31	10.69	2.19	2.15	1.25
Pre-decalcified slag	19.87	34.45	11.41	2.49	3.26	1.91

**Table 2:** Major chemical composition of vanadium-bearing steel slag (wt.%).

Chemical Composition	CaO	Fe_2_O_3_	MgO	Al_2_O_3_	V_2_O_5_	P_2_O_5_	MnO	TiO_2_	SiO_2_	Cr_2_O_3_	Other	Total
Content (%)	40.83	30.31	10.69	2.48	2.19	2.15	1.85	1.66	1.25	0.69	5.90	100

## Data Availability

The raw data supporting the conclusions of this article will be made available by the authors on request.

## Abbreviations

The following abbreviations are used in this manuscript:

XRD	X-ray Diffraction
SEM-EDS	Scanning Electron Microscope–Energy Dispersive X-ray Spectroscopy
ICP	Inductively Coupled Plasma Spectroscopy
L/S ratio	Liquid-to-Solid Ratio
XPS	X-ray Photoelectron Spectroscopy
